# Exploring the Interior of Europa with the Europa Clipper

**DOI:** 10.1007/s11214-023-00990-y

**Published:** 2023-08-25

**Authors:** James H. Roberts, William B. McKinnon, Catherine M. Elder, Gabriel Tobie, John B. Biersteker, Duncan Young, Ryan S. Park, Gregor Steinbrügge, Francis Nimmo, Samuel M. Howell, Julie C. Castillo-Rogez, Morgan L. Cable, Jacob N. Abrahams, Michael T. Bland, Chase Chivers, Corey J. Cochrane, Andrew J. Dombard, Carolyn Ernst, Antonio Genova, Christopher Gerekos, Christopher Glein, Camilla D. Harris, Hamish C. F. C. Hay, Paul O. Hayne, Matthew Hedman, Hauke Hussmann, Xianzhe Jia, Krishan Khurana, Walter S. Kiefer, Randolph Kirk, Margaret Kivelson, Justin Lawrence, Erin J. Leonard, Jonathan I. Lunine, Erwan Mazarico, Thomas B. McCord, Alfred McEwen, Carol Paty, Lynnae C. Quick, Carol A. Raymond, Kurt D. Retherford, Lorenz Roth, Abigail Rymer, Joachim Saur, Kirk Scanlan, Dustin M. Schroeder, David A. Senske, Wencheng Shao, Krista Soderlund, Elizabeth Spiers, Marshall J. Styczinski, Paolo Tortora, Steven D. Vance, Michaela N. Villarreal, Benjamin P. Weiss, Joseph H. Westlake, Paul Withers, Natalie Wolfenbarger, Bonnie Buratti, Haje Korth, Robert T. Pappalardo, The Interior Thematic Working Group

**Affiliations:** 1 Johns Hopkins Applied Physics Laboratory, Laurel, MD, USA; 2 Washington University in St. Louis, St. Louis, MO, USA; 3 Jet Propulsion Laboratory, California Institute of Technology, Pasadena, CA, USA; 4 CNRS, Nantes University, Nantes, France; 5 Massaschusetts Institute of Technology, Cambridge, MA, USA; 6 University of Texas at Austin, Austin, TX, USA; 7 University of California, Santa Cruz, Santa Cruz, CA, USA; 8 United States Geological Survey, Flagstaff, AZ, USA; 9 Georgia Institute of Technology, Atlanta, GA, USA; 10 University of Illinois Chicago, Chicago, IL, USA; 11 Sapienza University of Rome, Rome, Italy; 12 Southwest Research Institute, San Antonio, TX, USA; 13 University of Michigan, Ann Arbor, MI, USA; 14 University of Colorado Boulder, Boulder, CO, USA; 15 University of Idaho, Moscow, ID, USA; 16 German Aerospace Center Institute of Planetary Research, Berlin, Germany; 17 University of California, Los Angeles, Los Angeles, USA; 18 Lunar and Planetary Institute, University Space Research Association, Houston, TX, USA; 19 Cornell University, Ithaca, NY, USA; 20 NASA Goddard Space Flight Center, Greenbelt, MD, USA; 21 Bear Fight Institute, Winthrop, WA, USA; 22 University of Arizona, Tucson, AZ, USA; 23 University of Oregon, Eugene, OR, USA; 24 University of Texas at San Antonio, San Antonio, TX, USA; 25 KTH Royal Institute of Technology, Stockholm, Sweden; 26 University of Cologne, Cologne, Germany; 27 Stanford University, Stanford, CA, USA; 28 University of Washington, Seattle, WA, USA; 29 Alma Mater Studiorum – Università di Bologna, Bologna, Italy; 30 Boston University, Boston, MA, USA

**Keywords:** Europa Clipper, Interior, Subsurface ocean, Magnetic induction, Ice-penetrating radar, Tidal deformation

## Abstract

The Galileo mission to Jupiter revealed that Europa is an ocean world. The Galileo magnetometer experiment in particular provided strong evidence for a salty subsurface ocean beneath the ice shell, likely in contact with the rocky core. Within the ice shell and ocean, a number of tectonic and geodynamic processes may operate today or have operated at some point in the past, including solid ice convection, diapirism, subsumption, and interstitial lake formation.

The science objectives of the Europa Clipper mission include the characterization of Europa’s interior; confirmation of the presence of a subsurface ocean; identification of constraints on the depth to this ocean, and on its salinity and thickness; and determination of processes of material exchange between the surface, ice shell, and ocean.

Three broad categories of investigation are planned to interrogate different aspects of the subsurface structure and properties of the ice shell and ocean: magnetic induction, subsurface radar sounding, and tidal deformation. These investigations are supplemented by several auxiliary measurements. Alone, each of these investigations will reveal unique information. Together, the synergy between these investigations will expose the secrets of the Europan interior in unprecedented detail, an essential step in evaluating the habitability of this ocean world.

## Introduction

### Background

Prior to the space age, knowledge of Europa, or of any of the Galilean satellites, was due to telescopic studies. The Laplace resonance was known, along with general ideas of Europa’s size, mass, and surface reflectivity (cf. Alexander et al. 2009). The 1960s and 1970s introduced near-infrared reflectance spectroscopy, with the foundational detection of water ice on Europa’s surface (Moroz 1965; Johnson and McCord 1971), along with the first spacecraft observations of the jovian system by Pioneers 10 and 11, in 1973 and 1974, respectively. The principal contribution of the latter, through low-resolution imaging and Doppler radio tracking, was the refinement of Europa’s size and mass, and hence its density (nearly 3000 kg m^−3^; Smith 1978). The Voyager missions that followed the Pioneers across the asteroid belt employed highly capable, three-axis stabilized spacecraft to revolutionize our view of Europa and its Galilean siblings, transforming them in a matter of days, in March (Voyager 1) and July (Voyager 2) 1979, from objects of astronomical curiosity into fully fledged geological worlds (Smith et al. 1979a,b). Indeed, the modern era of icy (and other) satellite science was born in that year.

Europa was revealed to be a bridge world between the inner, hyper-volcanically active Io, and the larger, icy but more heavily cratered Ganymede and Callisto (Lucchitta and Soderblom 1982). Even though it was the least observed of all the Galilean satellites due to the specific trajectories chosen (closest approach distance of Voyager 2 was 206,000 km), Europa was clearly very lightly cratered with a surface rich in water ice. It did not possess dark surface units similar to those on Ganymede and Callisto, but exhibited instead “discolored,” reddish-brown mottled regions as well as a dense, intricate network of dark lineaments interpreted as traces of tectonic faults and fractures of uncertain character. The mottling was seen to be more widespread and intense on Europa’s trailing hemisphere, which came to be understood as being due to the implantation of sulfur atoms from the Io torus (Johnson et al. 2004; Carlson et al. 2009); this would not be the last time Europa’s interaction with the Jovian magnetosphere would prove important. Additionally, the satellite’s topography was observed to be very smooth at the kilometer scale (Steinbrügge et al. 2020b), certainly much smoother than that of Earth’s moon. In all, Europa represented an enigmatic but certainly geologically youthful icy satellite.

In terms of knowledge of its interior, Europa’s radius, volume, and mass were precisely determined (to <1%; Burns 1986). Given its surface composition and bulk density (considered to be identical to its uncompressed density), it was natural to think of Europa as being differentiated, possessing an ice shell overlying a rocky interior. The thickness and physical state of this ice shell were the subject of several contemporary studies (e.g., Fanale et al. 1977; Cassen et al. 1979, 1980; Ransford et al. 1981; Finnerty et al. 1981; Wilson and Head 1984). It can be noted that the paper of Squyres et al. (1983) was remarkably prescient. This work took as its cues the concept of tidal heating, similar to that occurring in Io, though diminished greatly in intensity, and an improved understanding of subsolidus convection (notably, still a work in progress), to argue for a subsurface ocean beneath a floating ice crust some ∼15 km thick. This hypothesis could not be proven with the data in hand, but the idea resonated, especially with the discovery (also in 1979) of deep-sea vent communities on Earth (e.g., Corliss et al. 1979), well below the ocean’s photic zone. For the first time, serious consideration of extraterrestrial life moved beyond Mars, where it had been parked since Lowell’s time, to an icy satellite in the outer solar system.

The Galileo mission to Jupiter, the successor mission to the Voyagers, revealed that Europa is truly an ocean world. Galileo explored the Jovian system between 1995 and 2003, and made discoveries that dramatically increased our knowledge of Europa, with important implications for its habitability. After several passes of Europa, Galileo was able to recover Europa’s quadrupole gravity term, C_22_, indicating that Europa was a fully differentiated body (ice/water shell over rocky mantle over metallic core; Anderson et al. 1998). But it was the Galileo magnetometer experiment in particular that provided the strongest evidence for a salty subsurface ocean beneath the ice shell, likely in contact with the rocky mantle (Kivelson et al. 2000). Galileo also revealed the geological complexity of Europa in full. The mean age of its sparsely cratered surface was determined to be between 40 and 90 Myr (Bierhaus et al. 2009), with several features indicating more recent if not ongoing activity, while numerous geological analyses pointed to heat flows sufficient to maintain a floating ice shell. Tectonic features were seen to be explainable by stresses arising from the tidal flexing of an ice shell that is mechanically decoupled from the deep interior (i.e., by a global subusrface ocean), but not by one that is grounded, and specific evidence has been presented for wholesale reorientations of the ice shell, by both non-synchronous rotation about the spin axis and true polar wander — both of which strongly suggest an ice shell decoupled from the deep interior (see Daubar et al. this collection). In sum, the gravity, magnetic induction, and tectonic evidence make a powerful case for the reality of Europa’s ocean. While an objective of the Europa Clipper mission is to confirm the existence of the ocean, the prime focus of the mission is to characterize this subsurface water layer and understand what it implies for habitability of this world and others like it.

In detail, Galileo’s Doppler tracking data were used to carry out several analyses of Europa’s gravity field. Anderson et al. (1998) reported on the analysis of the first four dedicated flybys (E4, E6, E11, and E12, according to the numbering scheme used by the Galileo project), combined with ground-based astrometric data and optical navigation observables from both Voyager and Galileo. By imposing the hydrostatic equilibrium constraint ($\mathrm{J}_{2}/\mathrm{C}_{22} = 10/3$ for a relaxed, slowly synchronously rotating, satellite), the degree-2 gravity field coefficients yielded, using the classic Radau-Darwin relation, a mean dimensionless Moment of Inertia (MoI) factor (C/MR^2^) of 0.346 ± 0.005 (Anderson et al. 1998), where $C$ is the polar MoI, and $M$ and $R$ are the mass and radius of Europa, respectively. Being substantially less than the 0.4 value for a uniform sphere, the conclusion was that Europa must be differentiated. Given the evidence for an icy surface and geophysical arguments for Europa’s likely internal thermal evolution, it was further concluded that Europa probably had a metallic core surrounded by a rock mantle and a water–rich outer shell in a liquid or solid state. An alternate analysis (Jacobson et al. 1999) used Galileo radiometric data up to flyby E19, together with Earth-based astrometry and Pioneer and Voyager radiometric and optical data, to produce quadrupole gravity coefficients for Europa values that were smaller (0.341 ± 0.002) than those published by Anderson et al. (though within the error bars of the original analysis). More recently, motivated by the new knowledge of the Jupiter system offered by the Juno mission, Gomez Casajus et al. (2021) presented a reanalysis of the Galileo tracking data acquired during the six best encounters with Europa, in terms of data quality and availability (flybys E6, E11, E12, E14, E16, and E19). The estimated quadrupole gravity field is compatible with hydrostatic equilibrium without imposing the a priori hydrostatic equilibrium constraint, as done previously. It should be noted, however, that the uncertainty in the gravitational flattening J_2_ is large. Moreover, the obtained C_22_ coefficient is slightly larger than in previous papers and yields a normalized mean MoI factor of 0.3547 ± 0.0024.

The static gravity field measurements are insensitive to the existence, much less the thickness, of Europa’s ocean and to the size and density of any metallic core (these being unconstrained by knowledge of the density and MoI alone). Magnetic induction results break the degeneracy regarding the existence of the ocean. As described in the review by Khurana et al. (2009), during five close passes by Europa, Galileo detected the electromagnetic signature of a conducting layer near Europa’s surface, one whose electric currents generated a dipole field approximately aligned with Europa’s orbit that cancelled out the variable component of the Jovian magnetic field, as well as other complex magnetospheric interactions. This cancelling field was not fixed in position or strength from pass to pass, but appropriate to the forcing Jovian field, and was too strong to have been due to either Europa’s ionosphere (not conductive enough) or a metallic core (too deep). The high conductivity that would be required of Europa’s upper rock mantle is simply unrealistic, leaving a conductive global ocean as the logical and most parsimonious explanation. Ongoing analysis (Tyler 2011; Vance et al. 2021a) suggests that flows within the ocean may influence the induction response, although this is a relatively small contribution from the total induced field.

### Objectives

One of the Europa Clipper’s Level-1 science requirements directly concern the interior of Europa. This requirement can be mapped to four broad science themes: characterization of the interior; confirmation of the presence of a subsurface ocean; identification of constraints on the depth to this ocean, and on its salinity and thickness; and determination of processes of surface–ice–ocean exchange. Three broad categories of investigation (augmented by several auxiliary measurements) are planned to interrogate different aspects of the subsurface structure and properties of the ice shell and ocean: magnetic induction, subsurface radar sounding, and tidal deformation. Alone, each of these investigations will reveal unique information. The synergy among these investigations will expose the structure and dynamics of the Europan interior in unprecedented detail, an essential step in evaluating the habitability of this ocean world.

The science requirements, themes, and planned measurements designed to address them are presented in Table 1, and the objective to characterize the ice shell and any subsurface water is discussed in detail below. In Sect. 2, we summarize the science investigations indicated in Table 1, with a focus on the particular measurements that will address the science questions. For more detail on these investigations, the reader is referred to the individual instrument papers. In Sect. 3, we discuss how the results of the individual investigations will be synthesized to reveal key parameters on the interior of Europa: the ice shell thickness, ocean thickness, and ocean salinity. Finally, in Sect. 4 we summarize how the integrated interior science will achieve the science objective of the Europa Clipper mission.

**Table 1:**
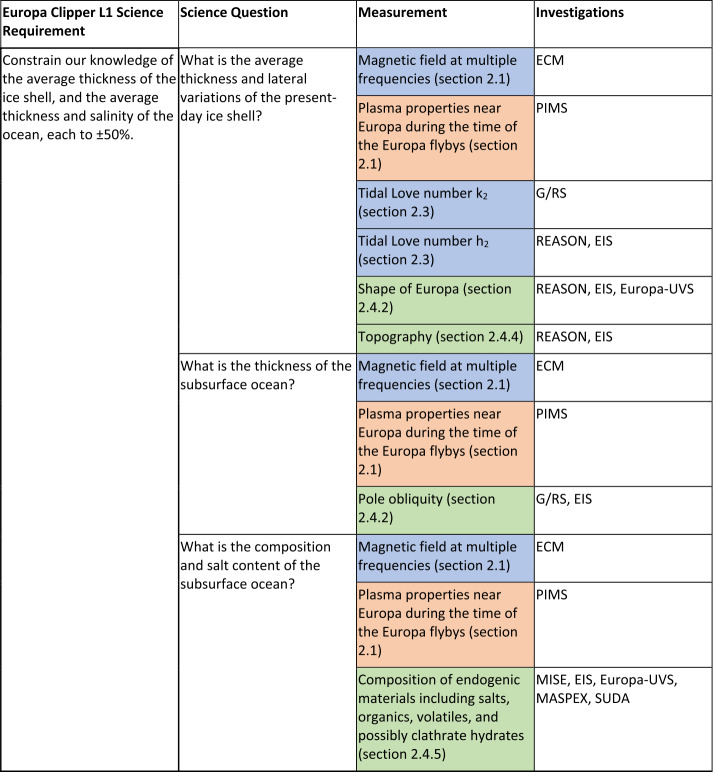

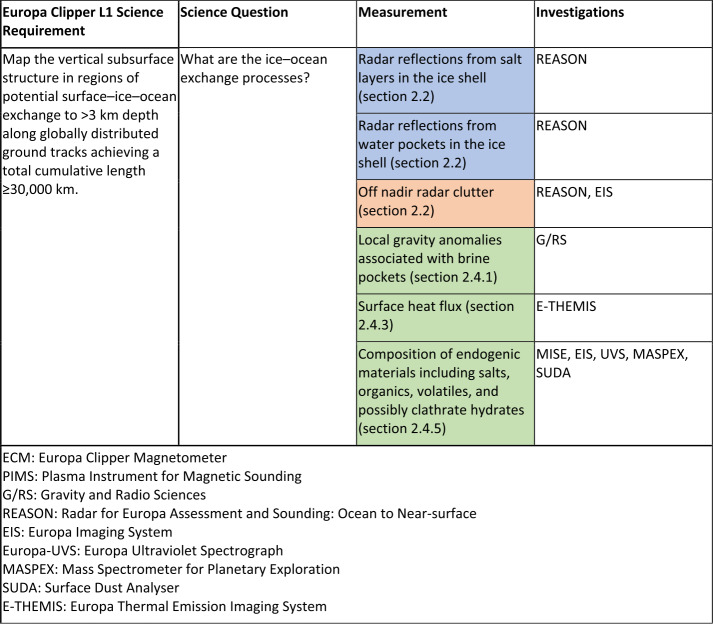
Interior Level-1 objectives and science questions

#### Ice and Ocean Properties

Gravity measurements performed by the Galileo spacecraft (Anderson et al. 1998) put constraints on the total thickness of the hydrosphere (ice plus ocean), initially estimated between 80 km and 170 km depending on assumptions on the rocky interior structure (Anderson et al. 1998; Sohl et al. 2002). Recent re-analysis of the Galileo data (Gomez Casajus et al. 2021) indicate that the hydrosphere may be thinner by 20–40 km than previous estimates, but the relative thickness of the ice shell still remains unconstrained. Geologic and geodynamic arguments predict that the ice shell thicknesses may range from a few km to >30 km based on mechanical, thermodynamic, cratering, and other methods (see Billings and Kattenhorn 2005, for a compilation). Although most interior models propose that ice shells several tens-of-kilometers thick at present are most probable (Howell 2021), ice shells thinner than ten kilometers cannot be ruled out, particularly if there is strong tidal heating in the rocky interior (Greenberg et al. 2002; Sotin et al. 2009). Although lateral variations in ice shell thickness at present are expected (Ojakangas and Stevenson 1989; Tobie et al. 2003; Nimmo et al. 2007; Ashkenazy et al. 2018), they cannot account for the wide range of thickness estimates published in the literature. This strong variability most likely represents a record of the ice shell thickness at different geological times (e.g., Figueredo and Greeley 2004; Leonard et al. 2018). Changes in the magnitude of tidal heating due to interactions with Io and Ganymede is indeed expected to affect the heat budget of Europa and hence the thermal equilibrium of its ice shell (Hussmann et al. 2002). The interaction between these moons takes the form of a resonance, in which Io (the innermost body) completes (almost) exactly four orbits in the time that Europa completes two, and Ganymede completes one. This 4:2:1 commensurability of the orbits is known as the Laplace resonance and is instrumental in maintaining the orbital eccentricity of Io and Europa, such that their orbits do not circularize, and tidal dissipation can heat their interiors over geologic timescales.

The equilibrium thickness of the ice shell depends not only on the heat production (tidal and radiogenic heating in the whole interior) but also on how internal heat is transported through the ice shell, either by conduction or sub-solidus convection (e.g., McKinnon 1999; Tobie et al. 2003; Moore 2006; Barr and Showman 2009; Allu Peddinti and McNamara 2019; Green et al. 2021; Howell 2021). Europa’s ice shell may have experienced multiple transitions between conductive and convective states (Mitri and Showman 2005), and it is unclear which state Europa occupies at present because predominantly convective and predominantly conductive ice shell solutions can both be used to explain current inferences and observations (e.g., Howell 2021). The objectives of the Europa Clipper mission include precise determination of the present-day average ice shell thickness and lateral variations, together with estimates of the near-surface thermal gradient. Knowledge of each of these quantities to an uncertainty of ±50% will provide key constraints on the internal heat budget of Europa and on the potential exchange between the ocean and the surface. Estimates of past ice shell thickness and thermal structure from geological interpretation will also be essential to reconstruct the hydrosphere evolution through time.

Magnetometer data from the Galileo mission confirmed the presence of a global saline liquid ocean underneath the icy shell (Khurana et al. 1998; Neubauer 1998; Kivelson et al. 1997, 1999, 2000). In principle, magnetic induction can provide constraints on the electrical conductivity, depth beneath the surface, and thickness of the ocean (e.g., Zimmer et al. 2000; Khurana et al. 2002; Schilling et al. 2007; Saur et al. 2010; Seufert et al. 2011). Hitherto, estimating these parameters individually has not been possible because the Galileo magnetometer investigation did not provide the temporal coverage of Europa’s induction response sufficient to infer signals at frequencies other than the primary frequency associated with Jupiter’s synodic period seen in the satellite’s rest frame. For instance, Schilling et al. (2007) found magnetic field data are best explained by electrical conductivity values of ≳0.5 S/m with ocean thicknesses of 100 km. However, these numbers are not uniquely diagnostic because other values rendering the same product of conductivity and ocean thickness agree with the measurements comparably well. Published estimates bound the electrical conductivity of the ocean between ∼0.3 and 3 S/m (Zimmer et al. 2000; Schilling et al. 2007), which results in large uncertainty in salt concentration estimates ranging from about 3 g/kg_H2O_ (brackish) up to 100 g/kg_H2O_ (hypersaline; Hand and Chyba 2007; Khurana et al. 2009). Observations at further inducing frequencies, such as those given by the orbital period of the moon or harmonics of Jupiter’s synodic rotation frequency, or the solar rotation rate will break the degeneracy between ocean conductivity, ocean thickness, and depth (e.g., Seufert et al. 2011; Vance et al. 2021a). Even with this additional information, the inferred ocean thickness and conductivity may have degenerate solutions. In addition to electric conductivity, the Europa Clipper mission will include identifying upper and lower bounds on ocean density from static gravity field and tidal monitoring. This will provide further constraints on the ocean composition and depth (e.g., Vance et al. 2018), complementary to magnetic induction. Joint inversion of geophysical and geochemical measurements are required to determine if Europa’s ocean is dominated by sulfates or is closer to Earth’s seawater or Enceladus’ ocean water composition, which has major consequences for the thermo-chemical evolution of Europa and the habitability of its ocean (Zolotov and Kargel 2009). Accounting for the temperature and pressure dependence of the electrical conductivity for candidate ocean compositions can help (Vance et al. 2021a).

Understanding the ice–ocean–floor interaction by identifying constraints on the distribution of salinity is a major science question of the mission (see Table 1, and Sect. 3.4). Although the presence of salts in tectonic and chaotic terrains (e.g., McCord et al. 1998, 2002; Dalton et al. 2005; Carlson et al. 2009; Shirley et al. 2010; Prockter et al. 2017; Trumbo et al. 2022) is indicative of material exchange with the subsurface, possibly with shallow liquid reservoirs that are perched in the ice shell, or with the ocean, estimates of the salt content in the ice shell remain poorly constrained, and the question of how representative these compositions are of the underlying ocean is a topic of vigorous debate (Kargel et al. 2000; Zolotov and Shock 2001; McKinnon and Zolensky 2003; Pappalardo and Barr 2004; Han and Showman 2005; Zolotov and Kargel 2009; Buffo et al. 2018, 2020; Vu et al. 2020; Vance et al. 2021b; Wolfenbarger et al. 2022a). Interpretation of Europa’s ocean composition and salt content in the ice shell based on the composition of its surface and ejecta grains is complicated by the speciation of surface salts due to freezing/refreezing process within the shell (Vu et al. 2016, 2020) and by radiation (e.g., Hand and Carlson 2015), as well as potential contamination by Io’s sulfur (see Becker et al. this collection, Sect. 6). Detection of salt-rich layers and brine lenses, if present inside the ice shell by ice penetrating radar techniques (Blankenship et al. 2009; Pettinelli et al. 2015; Schroeder et al. 2016; Kalousová et al. 2017), potentially correlated with fresh salt-rich deposits at the surface, will be essential to evaluate the salt content and how brine formation and migration contributes to the ice–ocean exchange processes in a variety of geodynamical contexts (e.g., Schmidt et al. 2011; Kalousová et al. 2016; Steinbrügge et al. 2020a; Chivers et al. 2021; Hesse et al. 2021). The bulk salinity of the ice shell will influence the strength of a radar reflection from a eutectic interface by governing the amount of thermodynamically stable brine (Culha et al. 2020). Large, saline reservoirs, if present, will represent strong radar reflectors and may also be potentially identified from magnetic induction signals and local gravity anomalies. Such measurements, combined with geophysical constraints on ice shell thickness, thermal structure, and oceanic composition mentioned above, will put fundamental constraints on the salt cycle on Europa and the chemical evolution of its hydrosphere.

#### Deep Interior

**Fig. 1:**
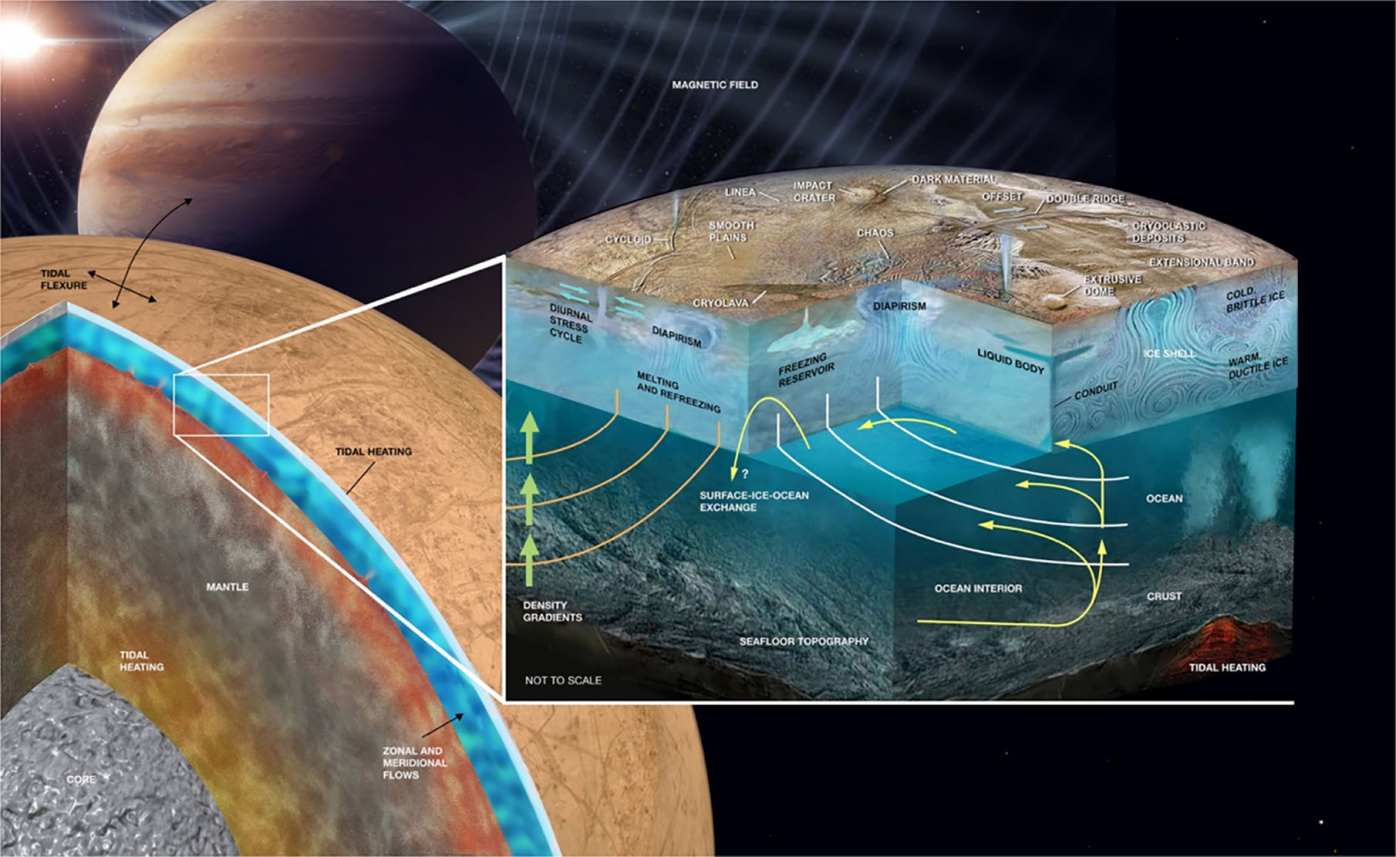
The left-hand side of the figure shows a cutaway view of Europa’s interior. From this image it is evident that despite the surficial appearance, Europa is not truly an icy moon. Rather, it is a rocky body covered in ice. The ice shell and underlying ocean form a thin (∼100 km thick) veneer of volatiles overlying a rocky mantle and metallic core. On this global scale, the key physical process that occur are the tidal dissipation in the lower ice shell and mantle, zonal and meridional flows in the ocean, and the induced magnetic field caused by the body’s passage through Jupiter’s variable magnetic field (shown in the background). Locally, most of the important features and processes occur in the ice shell and ocean, shown in the inset. Here, the ice shell is shown not as homogeneous, but highly variable. The cold brittle ice near the surface lies on top of warmer, ductile material below that is heated unevenly by tides. This may drive subsolidus convection in the ice shell resulting in upwelling ocean ice diapers, formation and re-freezing of melt lenses, and diurnal stresses. These processes manifest at the surface in the form of cycloids, double ridges, and chaos terrains

Our understanding of the deep interior of Europa comes primarily from Europa’s density and gravitational quadrupole coefficients inferred from the Doppler shift of Galileo’s radio communication signal during Europa flybys (Anderson et al. 1998; Sohl et al. 2002; Schubert et al. 2009) and from theoretical modeling. As illustrated in Fig. 1, Europa is thought to have differentiated into a metallic core, a silicate mantle, and a hydrosphere (Schubert et al. 2009). However, Europa’s bulk density and moment of inertia are the only gravitational parameters that constrain the density and radius of each layer, so the problem is underconstrained. Furthermore, the inversion of the C_22_ coefficient inferred from Galileo data to yield an estimate of Europa’s moment of inertia has in most studies relied on the assumption that the moon is relaxed to hydrostatic equilibrium. The actual value of Europa’s moment of inertia may be quite different, an uncertainty that the Europa Clipper gravity measurements will address (see Mazarico et al. 2023).

Although a two-layer model consisting of a homogenous rock/metal core, topped with a water/ice layer, could fit the existing gravity data, this would require the inner layer to have a density higher than 3650 kg m^−3^ (Gomez Casajus et al. 2021). This value is higher than the bulk density of nearby Io, ∼3528 kg m^−3^ (Schubert et al. 2009). However, it is worth remembering that Io’s interior is far warmer than Europa’s, and Io has lost much of its lighter volatiles. An enrichment in dense metallic phases relative to Io is unlikely, however, considering the origin of the Galilean moons (Becker et al. this collection). Furthermore, radiogenic heating in the silicates is expected to be sufficient to raise Europa’s interior temperatures enough to cause differentiation leading to the formation of a metallic core such as that found inside Ganymede (Greeley et al. 2004; Schubert et al. 2004, 2009). Sohl et al. (2002) estimated that the core radius may range between 0.1 and 0.45 × Europa’s radius depending on the assumed hydrosphere thickness and averaged mantle/core density. Recent re-analysis of the Galileo data (Gomez Casajus et al. 2021) suggests a thinner hydrosphere and less dense interior than initially estimated; the size, state, and even the existence of a metallic core on Europa could not be conclusively identified by previous studies. Improved and independent determination of J_2_ and C_22_ by Europa Clipper, as well as the pole obliquity, will yield more accurate constraints on Europa’s density profile, although large uncertainties will remain due to the large space of possible properties describing the core and mantle materials.

The size and composition of Europa’s rocky mantle is unknown. Based on the bulk density and moment of inertia constraints, the rocky mantle could comprise either hydrated or dehydrated silicates, or mixture of the two (Schubert et al. 2009). However, modest levels of heating (radiogenic or tidal) could dehydrate the mantle (Greeley et al. 2004; Schubert et al. 2009), especially if it is not convecting, unless a process exists to move water back into Europa’s rocky mantle (such as plate tectonics). The mantle is therefore expected to be mostly dehydrated except potentially in the uppermost 25-50 km of the mantle where hydrothermal circulation may occur (McKinnon and Zolensky 2003; Vance et al. 2007; Travis et al. 2012). A cooler thermal evolution pathway is possible if a large fraction of the potassium (and thus ^40^K) originally in the rock was leached during an early phase of aqueous alteration (Engel et al. 1994; Castillo-Rogez and Lunine 2010).

If an early episode of heating resulted in partial melting of the mantle, the formation of a silicate crust is expected, partitioning radiogenic material into the crust and limiting present day heat sources in the rocky mantle (Moore and Hussmann 2009; Běhounková et al. 2021). The likelihood of present-day activity in Europa’s rocky mantle is of great interest, because hydrothermal activity at the seafloor could provide a source of energy for life (Hand et al. 2009; Vance et al. 2016) and enhance mixing within the ocean that would promote rock-ocean-ice exchange necessary for redox reactions (e.g., Soderlund 2019). If the top of Europa’s silicate crust is thermally and mechanically similar to the surface of Io, the expected mantle heat flux would be roughly 100 mW m^−2^ (Greenberg et al. 2002) and would produce a conductive ice shell roughly 6 km thick. On the other hand, a cold, stiff mantle would produce no tidal heat. In reality, thermal-orbital coupling could have resulted in Europa oscillating between hot and cold mantle states (Hussmann and Spohn 2004). Still more recent work (Běhounková et al. 2021) demonstrated that a combination of radiogenic heating and tidal dissipation could result in magma production throughout much of Europa’s history.

Although these models are instructive, they are not conclusive. No observational evidence exists that supports or refutes the existence of a source of heat or fresh rock at Europa’s seafloor. The objectives of the Europa Clipper mission include the acquisition of a combination of geophysical measurements (e.g., gravity data, Mazarico et al. 2023), chemical analysis, and thermal images of recently active areas on Europa’s surface and any associated plumes (see Becker et al. this collection), which may imply present-day activity on Europa’s seafloor and/or a warm interior.

#### Exchange Processes

Heat flow into the ocean from the seafloor combined with heat loss through the overlying ice shell is expected to drive thermal convection globally in the ocean, modulated by compositional buoyancy associated with salinity gradients that may enhance the vigor of convection (positive gradient) or have a stabilizing effect (negative gradient) (e.g., Soderlund et al. 2014; Ashkenazy et al. 2018; Soderlund 2019; Soderlund et al. 2020; Wong et al. 2022). Ocean flows may also be driven mechanically through tides, libration, and/or precession (e.g., Lemasquerier et al. 2017) and electromagnetically through interactions with Jupiter’s magnetosphere (Gissinger and Petitdemange 2019). This global ocean circulation may be influenced locally by hydrothermal plumes rising above seafloor hotspots, if any (e.g., Goodman et al. 2004), or potentially the release of brines from the ice shell due to englacial melting events (e.g., Nimmo et al. 2002; Sotin et al. 2002; Schmidt et al. 2011) and associated draining to the ocean. By modulating the heat flux at the ice–ocean interface, the oceanic circulation is expected to influence the interface evolution and hence the ice shell dynamics. In return, melting and freezing along the ice–ocean interface should create salinity gradients, which may drive meridional currents if the ice thickness varies from pole to equator (Zhu et al. 2017; Ashkenazy et al. 2018). Vertical gradients in salinity, for example due to changing ice thickness, may lead to double-diffusive convection within the bulk ocean of potential interest for creating energetic niches for life (Vance and Goodman 2009). However, numerical models indicate that such interfaces (also known as thermohaline staircases) dissipate relatively quickly, possibly within 10 kyr after the source/sink for salinity disappears (Travis et al. 2012), though other circumstances may prolong a double-diffusive state for geologically significant periods (Wong et al. 2022).

The objectives of the Europa Clipper mission include acquiring knowledge of the global exchange processes between the ocean and ice shell, and on the pattern of oceanic heat flux as has been done for Titan and Enceladus (Kvorka et al. 2018; Čadek et al. 2019). Estimates of lateral variations in ice–ocean interface depths, using gravimetric, topographic, magnetic, and radar sounding techniques, will be essential towards identifying these constraints. Radar observations may also be able to detect regions of accreted marine ice to further test ocean circulation and heat flow hypotheses (e.g., Blankenship et al. 2009). Preliminary assessments further suggest that oceanic flows may be constrained from the magnetic induction response (Tyler 2011; Vance et al. 2021a), which would put key constraints on the oceanic dynamical regime.

The variety of landforms observed on Europa’s surface are specific records of the internal evolution processes and lithospheric structure at the time of formation (e.g., Doggett et al. 2009; Nimmo and Manga 2009; Schenk and Turtle 2009). Each type of landform (ridges, bands, pits, domes, chaos, impacts, etc.) implies specific local stress and thermal conditions in the ice shell. The objectives of the Europa Clipper mission include comprehensive mapping of the surface landforms and understanding of the tectonic and cryovolcanic history of Europa. The interpretation of individual landforms using both surface/subsurface observations and theoretical models (e.g., Howell and Pappalardo 2018) can provide insights on the local mechanical structure of the ice shell as well as on the geodynamical process at their origin. Global stratigraphy (e.g., Figueredo and Greeley 2004; Doggett et al. 2009), using much better coverage than was achieved with Galileo, will also allow for a detailed assessment of tectonic resurfacing and chaos terrain formation sequences, essential to reconstructing the geodynamical history of the ice shell and the implications for exchange processes (see Daubar et al. this collection, for more details). In particular, detailed mapping and resulting tectonic reconstruction may reveal areas of surface material recycling (e.g., Mével and Mercier 2005; Kattenhorn and Prockter 2014; Culha et al. 2014). Detailed geophysical and geological investigation of such identified areas will permit testing whether crustal recycling zones on Europa mostly result from cold Earth-like subduction processes (Kattenhorn and Prockter 2014; Johnson et al. 2017) or are rather associated with internal melting processes and brine drainage (e.g., Vance et al. 2021b); and whether sills can explain the origin of domes and lenticulae (Michaut and Manga 2014). Understanding the context of surface recycling on Europa is essential to constraining the dynamics of the ice shell (Howell and Pappalardo 2019) and to assessing the amounts of surface material that might be recycled in the ocean (e.g., Vance et al. 2016). Maps of surface temperature variation from infrared surface imaging (E-THEMIS), combined with geomorphology determined from visible imaging (EIS) and compositional variation (MISE) may also indicate the relative age of surface features and inform turnover and exchange rates (Hayne et al. 2017) (see Becker et al. this collection, for more details). Detection of shallow structures and liquid water and brines by ice-penetrating radar could support evaluation of hypothesized surface–ice exchange mechanisms (Blankenship et al. 2009), and spatial variation in sounding depths may differentiate zones of relatively colder (downwelling) ice indicative of convection (McKinnon 2005; Kalousová et al. 2017).

The origin of the putative eruptive surface plumes identified in Hubble observations (Roth et al. 2014; Sparks et al. 2016, 2017; Giono et al. 2020) and in Galileo magnetometer and plasma wave data (Jia et al. 2018) is unclear. If Europa’s plume material originates in the subsurface ocean, this would suggest a complicated, subsurface plumbing system. As stress states in the ice shell may not allow for fractures to extend directly from the surface to the subsurface ocean, plume material may transfer to the surface in a series of fractures that are connected to one or more discrete fluid pockets in the ice shell (Muñoz-Iglesias et al. 2014). Such a configuration would be similar to terrestrial magmatic systems, in which magma is rarely transported from its source to the surface for eruption along a single fracture or path (kimberlite pipes being a notable exception). Rather, terrestrial magmas travel a somewhat convoluted path in which fractures connect the magma source to many small magma pockets perched at increasingly shallow depths in the crust en route to the surface (e.g., see Cashman et al. 2017, and references therein). Plumes may also have relatively shallow sources (Daubar et al. this collection), such as subsurface lenses below chaos terrains (e.g., Schmidt et al. 2011) and associated with cooling impact craters (Steinbrügge et al. 2020a). An objective of the Europa Clipper mission is to search for and investigate these plumes in order to constrain the properties of their source(s). The Europa-UltraViolet Spectrometer (Europa-UVS) will conduct a plume search. The mass spectrometer (MASPEX) will constrain the timing and scale of chemical fluxes in the mantle-ocean-ice–shell system. Sampling of plumes and fresh surface deposits (by MISE, Europa-UVS, and SUDA) can be used to evaluate potential ocean compositions. Although there are clear implications of the potential plume sources for interior science, the focus of the plume investigation is on current activity. This is described in more detail in Vance et al. (this collection).

## Investigations

The science questions for Europa’s interior science are highly interdisciplinary and cannot be answered with a single investigation. Fortunately, Europa Clipper carries a highly diverse payload of ten instruments plus a gravity and radio science investigation enabled by the telecommunications subsystem that target the objectives in an integrated manner. Three investigations in particular are focused on interior science, making observations that will constrain the ice shell thickness, ocean thickness, and ocean salinity, as well as illuminating the exchange processes between the ocean and the surface. The magnetic induction experiment using the Europa Clipper Magnetometer will result in coupled solutions for the thickness and the conductivity of the subsurface ocean. The sounding experiment using the Radar for Europa Assessment and Sounding: Ocean to Near-surface (REASON) instrument will constrain the thickness of the ice shell, and map the vertical subsurface structure, as well as constrain its tidal deformation. Observations of the tidal deformation of the ice shell from the gravity and radio science (G/RS) investigation will recover the combined strength and thickness of the ice shell. These three investigations are supported and supplemented by several other investigations from the entire payload that will be used to resolve ambiguities and degeneracies.

**Fig. 2:**
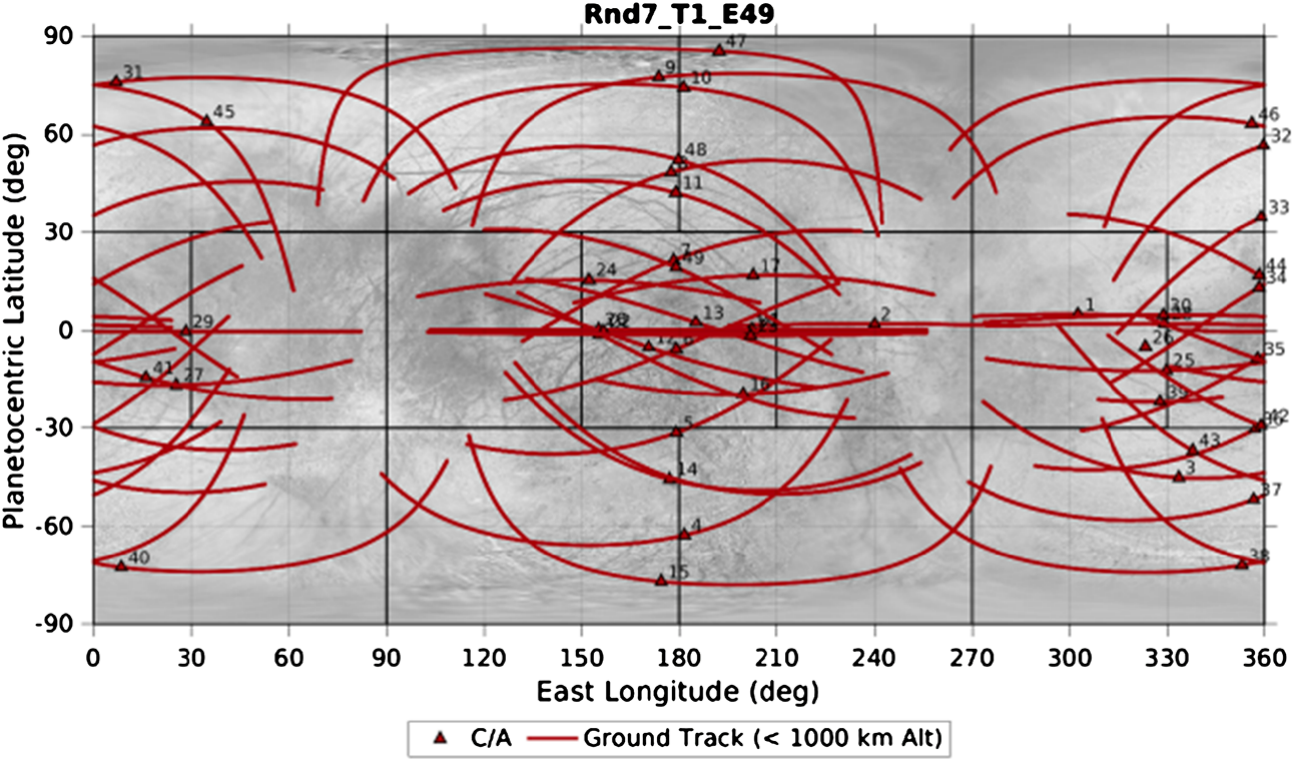
Map of Europa from USGS (Image source: https://astrogeology.usgs.gov/maps/europa-voyager-galileo-global-mosaics) with Europa Clipper groundtracks and closest approach points for the baseline trajectory at the time of publication (Rnd7_T1_E49)

In addition to the instrumentation, the investigations are enabled by Europa Clipper’s unique trajectory. Rather than placing the spacecraft in orbit about Europa, where it would be subject to an inhospitable radiation environment, it enters a highly eccentric orbit about Jupiter, and spends most of its time at a larger (and safer) distance from the planet. The Europa Clipper will execute 49 close flybys of Europa itself, with closest approach distances less than 1000 km, and sometimes within 25 km of the surface. This tour design has multiple advantages over an orbital campaign. Less propellant is needed to enter a Jupiter orbit than an orbit about Europa itself. The spacecraft is able to make several encounters much closer to the surface than could be safely done while in orbit. By spending most of its time outside the high radiation environment near Jupiter, the mission lifetime is extended substantially; the baseline tour is 4.3 years long. Finally, the investigations will be able to characterize the ambient Jovian plasma and magnetic environment far from Europa, so that the observations at Europa can be placed in the context of the larger system. The tour has been designed to provide robust geographic coverage. An example of the distribution of close flybys is shown in Fig. 2. For more details on the mission design, please see the Mission System Overview paper in this collection.

In this section, we summarize the key contributions of each investigation to the interior science, and in the following section we discuss how the disparate datasets are synthesized into a coherent story about Europa’s interior.

### Magnetic Induction

A magnetic field that varies in time generates a curling electric field in accordance with Maxwell’s law of induction. In the presence of a conducting material such as a salty ocean, this curling electric field will drive electric currents that generate a secondary, induced magnetic field that opposes the variation in the external field. When the body has spherical symmetry and the oscillating external magnetic field is uniform across it, the induced magnetic field consists of a dipole oscillating in response to the driving field (e.g., Saur et al. 2010). The magnitude and phase delay of the induced field are determined both by the radial conductivity structure of the body and the frequency of the oscillating external field, providing a means to probe Europa’s interior. For example, a perfectly conducting layer generates the maximum induction response, fully excluding the time-variable field from the interior of the conductor. The maximum induction amplitude therefore occurs at the pole of the induced dipole and is equal to the amplitude of the oscillating external field. In this idealized case, the observed induction field reveals the distance from the spacecraft to the surface of the conducting layer, enabling determination of the thickness of the non-conducting ice shell. On the other hand, measuring the induced field from a perfect conductor provides no information on the thickness of the conducting, liquid water layer. In contrast to this idealized case, actual oceans have finite conductivity and yield smaller induction amplitudes with a phase delay between the external and induced fields, but the dependence on Europa’s internal structure is generally not unique—many plausible oceans can produce the same induction signature. To break this degeneracy and characterize the subsurface ocean, Europa Clipper will observe the induced response at multiple frequencies.

**Fig. 3:**
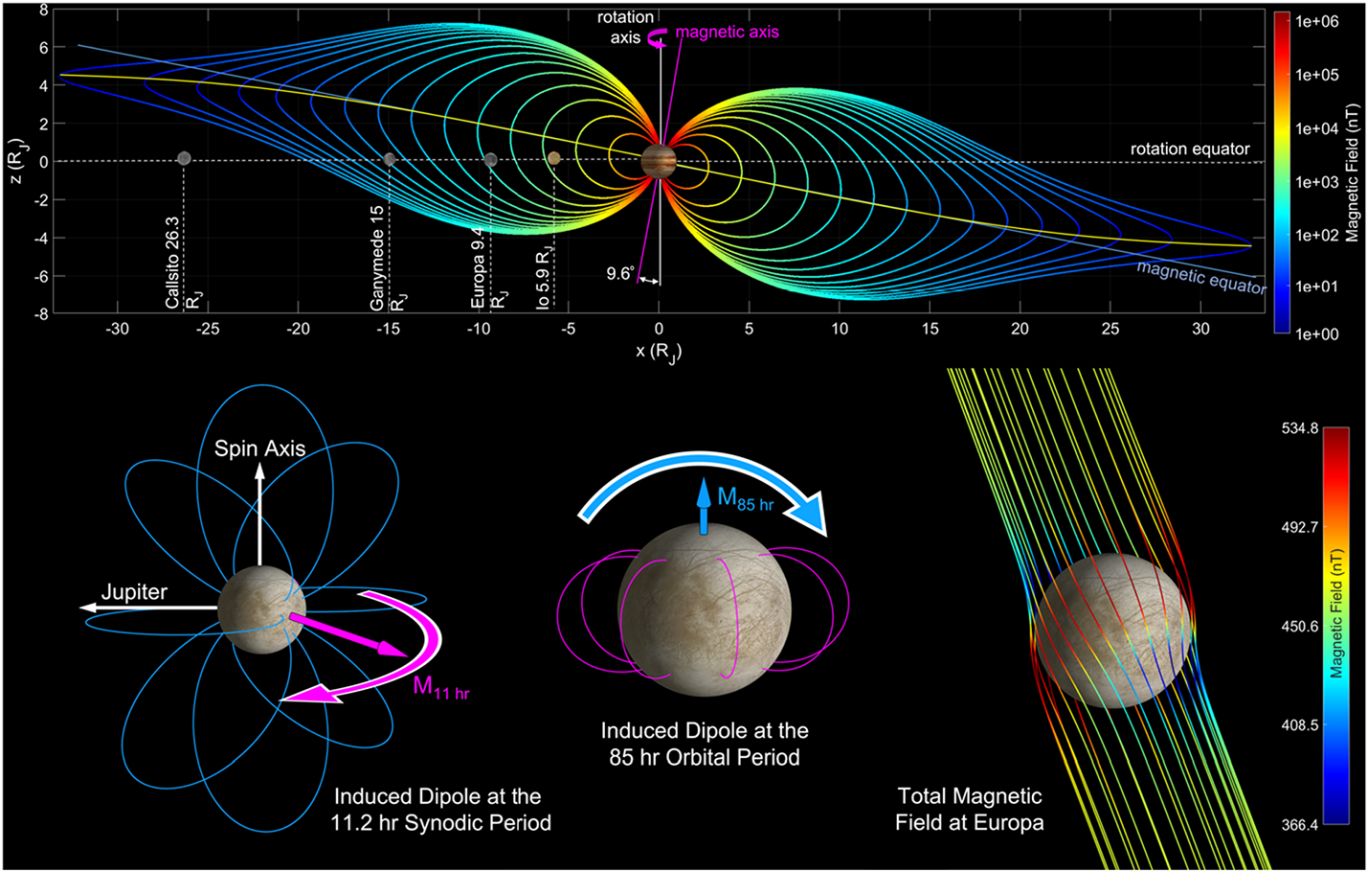
Geometry of Jovian magnetic field and induced magnetic field at Europa. (Top) As Jupiter rotates, the magnetic field in Europa’s frame varies at the 11.2 hour synodic period of Jupiter (i.e., the time required for Jupiter to return to the same geographic longitude as observed from Europa) due to the 9.6° tilt of Jupiter’s magnetic axis with respect to its rotation axis. (Bottom left) Because the synodic variation is primarily confined to Europa’s orbital plane, the induced magnetic moment, and the associated dipolar field, rotates approximately in Europa’s equatorial plane at the 11.2 hour period. (Bottom middle) Europa’s orbital eccentricity causes additional variation in the magnetic field at its 85 hour orbital period, which generates an induced magnetic moment, and associated dipolar field, approximately aligned with or against Europa’s spin axis. (Bottom right) The total magnetic field at Europa consists of Jupiter’s strong time-varying magnetic field and Europa’s induced dipolar magnetic field at multiple frequencies. Not pictured is the magnetospheric plasma corotating with Jupiter near the magnetic equator, which sweeps past the moon from the trailing side and complicates the interpretation of the measured magnetic field

The external magnetic field at Europa varies at multiple frequencies as a result of Jupiter’s rotation, Europa’s orbital motion, and the interaction of the Jovian magnetosphere with the solar wind (e.g., Seufert et al. 2011). The largest variation (∼200 nT) occurs at the 11.2 h synodic period, principally due to the 9.6° tilt of Jupiter’s dipole axis with respect to its spin axis. The Galileo spacecraft recorded the induced dipole produced in response to this variation (e.g., Khurana et al. 1998; Kivelson et al. 2000), but owing to the degeneracy described above, subsequent work has only succeeded in bounding the possible ocean parameters (e.g., Hand and Chyba 2007; Khurana et al. 2009). However, variations at the orbital (85.2 h, ∼20 nT) and second harmonic of the synodic period (5.6 h, ∼20 nT) are expected to produce appreciable induction responses, which can break the single-frequency degeneracy, revealing a unique combination of ice shell thickness, ocean thickness, and ocean conductivity that fits the data (Khurana et al. 2002; Seufert et al. 2011; Vance et al. 2021a). The geometry of the Jovian magnetic field and the induced field at Europa are illustrated in Fig. 3.

These measurements will be performed by the Europa Clipper Magnetometer (ECM), which consists of three fluxgate magnetometers mounted on an 8.5-m-long boom. Over the course of the mission, ECM measurements of Jupiter’s magnetic field far from Europa (≳3 R_Europa_) will be combined with data taken during close approaches to determine the driving and induced field amplitudes for at least two frequencies with a precision of ±1.5 nT. For the expected driving field amplitudes and plausible ocean structures, this is sufficient to characterize Europa’s internal structure (see Biersteker et al. 2023; Kivelson et al. this collection). Comparing ECM measurements with more complex models of Europa’s internal structure could, in principle, offer a more detailed description of the Europan ocean. These models may include complex radial conductivity structure informed by the bulk properties, geophysics, and laboratory measurements of materials under pressure, the magnetic signature of ocean currents (Tyler 2011; Vance et al. 2021a), and the effect of asymmetry in the overlying ice shell (Styczinski et al. 2022). An even more granular picture of Europa’s interior can be obtained by combining magnetometry with complementary measurements obtained by the gravity investigation, which can constrain Europa’s density structure, and REASON’s radar sounding, which can bound the ice shell thickness.

Fruitful analysis of the induced magnetic fields from the interior of Europa, however, requires constraining other contributions to the magnetic field perturbations around Europa. These contributions can stem from magnetic field perturbations in the magnetospheric field of Jupiter, magnetic fields caused by the interaction of magnetospheric plasma with Europa’s ionosphere, and induced magnetic fields in Europa’s ionosphere (Schilling et al. 2007; Saur et al. 2010). Consequently, observations of the magnetic field near Europa must be corrected for these effects. The distribution of plasma near Europa is spatially and temporally variable and is not well-constrained by existing measurements or models. Few ionospheric observations exist—just ten electron density profiles from Galileo radio occultations (Kliore et al. 1997; McGrath et al. 2009). Due to the temporal variability of the interaction, the plasma properties need to be determined during the times of the Europa flybys.

Measurements and modeling by Europa Clipper will provide these required corrections for plasma effects. During each flyby, the PIMS instrument (Westlake et al. this collection) will provide the plasma density, velocity and temperature moments for low-energy ion species and electrons, while ECM monitors the ambient magnetic environment. Each flyby transects the plasma interaction region, sampling the upstream environment, Alfvén wing structure, and Europa’s ionosphere. These measurements serve as the basis for inputs into multi-fluid (ions and electrons) magnetohydrodynamic (MHD) models, such as that of Harris et al. (2021), which self-consistently simulate the plasma interaction at Europa. The magnetic perturbation arising from the moon-plasma interaction can be estimated from these MHD models and subsequently subtracted from the ECM data to isolate the induction signal. In addition to PIMS, REASON will measure the total electron content along the line of sight between the spacecraft and the surface of Europa (Grima et al. 2015; Scanlan et al. 2019; Peters et al. 2020), while radiometric Doppler observations made by the spacecraft’s radiofrequency subsystem during occultations of the Earth by Europa will result in total electron counts along the line of sight (Park et al. 2011; Mazarico et al. 2023). The aggregated electron density measurements from REASON and ion density from PIMS, when compared to the ionosphere simulated by the MHD model, provides a means of model validation. For a more in-depth description of how plasma effects will be accounted for in the magnetometer data see Kivelson et al. (this collection).

### Subsurface Sounding

Cold ice is largely transparent at radio frequencies, a fact that has allowed for the radar sounding of ice masses on Earth and Mars (Schroeder et al. 2020; Picardi et al. 2005). Derived from this heritage, the REASON radar sounder on Europa Clipper will characterize the vertical structure of the ice shell and surface–ice shell-ocean exchange, constrain the depth of the ice–ocean interface, and determine the structure of the near-surface (Blankenship et al. 2009; Daubar et al. this collection). We address the first three goals here.

Unlike other investigations on Europa Clipper, REASON is an active remote sensing system, which generates its own radio-frequency photons. Energy must propagate out and back through space, the surface, and into the subsurface medium, and returning echoes are recorded. Given the large dimensions of radio wavelengths (1–100 m for the HF and VHF bands), REASON must record individual echoes coherently (i.e., preserve phase and amplitude information) to meet resolution requirements. After the data are downlinked, these echoes are summed on the ground through a process known as coherent integration or “stacking”, which increases the signal to noise ratio (Peters et al. 2005).

Radar waves reflect from interfaces marking contrasts in dielectric permittivity, but they are attenuated by the electrical conductivity of the ice. For subsurface echoes, an ice–water interface will reflect over 75% (−1 dB) of incoming energy, while a cold salt–ice interface (an example of ice shell structure) will reflect less than 1.5% (−18 dB). However, as the ice is warmer (and more electrically conductive) near an ice–water interface, the salt–ice interface echoes can easily appear brighter to radar than the ice–water interface due to attenuation by the overlying warm ice. To resolve this ambiguity in echo strength in the context of unknown signal attenuation, complementary data are required, especially altimetry, to determine whether interfaces are in flotation over a liquid. From REASON altimetry profiles, we can predict (for a given buoyancy contrast) the shape of the subsurface interface. This approach requires minimum ground track lengths depending on the target; for icebergs in the shallow subsurface (based on typical lengths scales seen in the Conamara chaos terrain on Europa), 10 km length scales should be sufficient; testing for the ice–ocean interface requires hemispheric-scale ground tracks. REASON is also sensitive to surface and volume scattering, which is a function of wavelength, surface roughness, and subsurface structure. For a given radar wavelength, roughness or subsurface porosity at that scale can degrade the coherence of the returning energy.

REASON uses two complementary radar arrays: a low resolution 9 MHz high frequency (HF) array for sounding that is relatively insensitive to surface and volume scattering and that is intended to penetrate more reliably into Europa’s ice crust, and a 60 MHz very high frequency (VHF) dual-channel interferometric array for both sounding and altimetry with high resolution. The latter is intended to limit the impact of Jovian radio-frequency noise and plasma dispersion, but it is more sensitive to scattering losses (see as described in the REASON investigation publication in this collection). The HF and VHF echoes reflected from Europa and received by the REASON instrument are organized into 2-D slices of detected energy that are termed “radargrams”; where the X-axis represents the along-track direction and the Y-axis represents time delay relative to signal transmission. With appropriate corrections to account for different speeds of propagation inside different media, depth images can be derived (Blankenship et al. 2009).

For these radargrams, an important complication for interpretation for any orbiting sounder is off-nadir “clutter” echoes with the same time delay as nadir subsurface echoes directly below the spacecraft, but coming from discrete targets that actually arrive at the spacecraft at an angle. Europa Clipper employs five approaches for dealing with clutter. For along-track clutter, REASON must collect enough coherent echoes to track the changing distance to surface scatterers at the wavelength scale as it passes overhead, allowing echoes only from directly below the spacecraft to be selected (Scanlan et al. 2021).

The discrimination of clutter from the side (cross-track clutter) requires one of the other approaches. For the deep ice shell, where resolution requirements are low, REASON reprojects its radargrams assuming all observed echoes are reflections from cross-track surface clutter. By comparing this reprojection with global surface mosaic images that will be collected by the EIS camera system, echoes that align with surface topographic features can be discriminated (Holt et al. 2006). For the shallow ice shell, it is possible to produce “cluttergrams” predictions of what the surface generated energy looks like from the perspective of REASON (Ferro et al. 2012), using stereo digital elevation models (DEMs) generated by the EIS Wide Angle Camera (WAC; Turtle et al. this collection).

In the absence of DEMs, off-nadir clutter can also be discriminated from shallow subsurface echoes using the interferometric capacity of REASON’s VHF band (Castelletti et al. 2017; Haynes et al. 2018a,b, 2020). The phase difference between echoes received by the VHF antennas on either solar array can be tracked, and compared to the phase difference of echoes expected to emanate from directly below the spacecraft. To test hypotheses for individual features in the radargram, this phase difference is compared with the predicted phase difference for surface clutter; and the features are thus determined to be at nadir and at depth, or off nadir and to the side. Lastly, the trajectory is designed such that each ground track has multiple intersections. As radar is highly anisotropic, the different directions over the same region can be very revealing. With the combination of WAC DEM data and interferometry, hypotheses regarding the complex 3D structure of Europa’s landforms may be inferred.

### Tidal Deformation

As Europa travels along its eccentric orbit, the tidal forcing imposed by Jupiter on Europa varies in magnitude, which is reflected in Europa’s response. The tidal response takes the form of deformation of Europa’s surface and internal redistribution of mass. The degree of density distribution is characterized by the gravitational Love number $k$, which can be described as the ratio of the gravitational potential arising from the tidal bulge to the tidal forcing itself (in this case the gravitational potential due to Jupiter as seen from the surface of Europa). Of particular interest is $k_{2}$ the Love number at spherical harmonic degree 2, corresponding to the dominant wavelength of the tidal potential. The magnitude of $k_{2}$ is a function of the effective rigidity of the entire body. An infinitely rigid body would not deform at all in response to tidal forcing, and $k_{2}$ would be zero. A uniform fluid body would deform with no resistance for a maximum $k_{2}$ of 1.5. A more realistic body would have intermediate values. Because $k_{2}$ depends on the interior structure, its measurement by Europa Clipper can provide constraints on the physical state of Europa, particularly the ocean and ice shell. The presence or absence of a subsurface ocean is a strong control on the magnitude of the Love number. An ice shell that is decoupled mechanically from the interior by a fluid layer will have a much larger $k_{2}$ than an ice shell that is locked onto the silicate mantle. Thus, the detection of a high value for the Love number ($k_{2} > 0.15$) would be conclusive evidence of the existence of an ocean (Park et al. 2011, 2015; Mazarico et al. 2015; Verma and Margot 2018). In the presence of an ocean, $k_{2}$ varies inversely with the thickness of the ice shell. Tidal deformation alone cannot provide a unique determination of the ice shell thickness, however, because the Love number is also a function of the rigidity of the ice shell and it is influenced by the ocean density (Mazarico et al. 2023). There is a tradeoff between these parameters for any value of $k_{2}$.

**Fig. 4:**
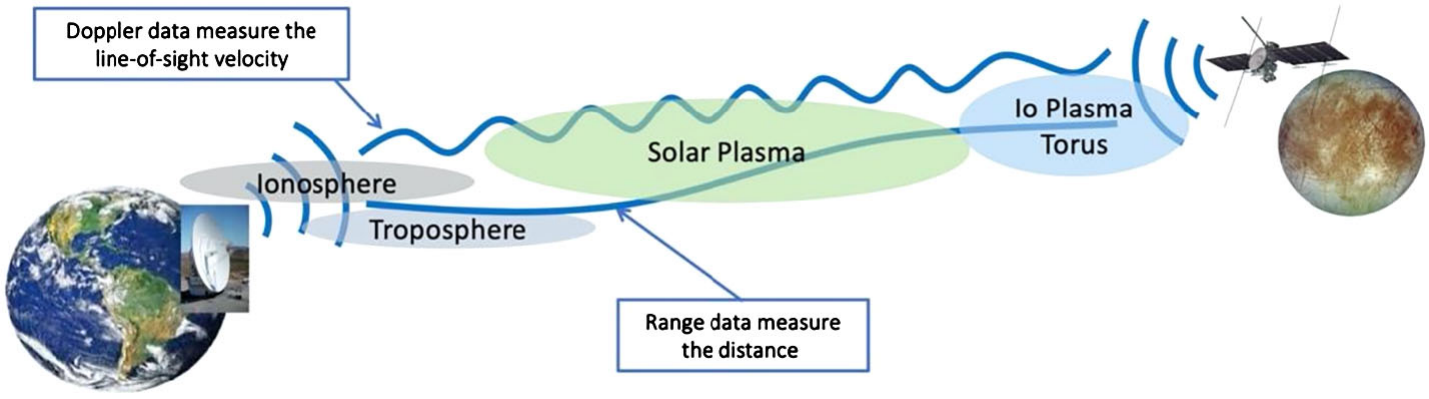
Graphical representation of the Doppler tracking of the Europa Clipper flight system using the DSN, including sources of radio noise

The time variations of Europa’s gravity field at degree 2 can be determined by accurately tracking the Europa Clipper spacecraft at multiple points along its orbit as it flies by Europa, that is, over a range of orbital phases. As illustrated in Fig. 4, radio signals from the NASA Deep Space Network (DSN) received and retransmitted by the spacecraft communication subsystem enable precise observations of the Doppler-shifted signal frequency, and thus of the spacecraft line-of-sight velocity with respect to the radio stations on Earth. This technique was successfully used by the Cassini mission to determine the long-wavelength static gravity field of several icy satellites of Saturn (Thomas et al. 2007; Mackenzie et al. 2008; Iess et al. 2010, 2014; Tortora et al. 2016: Zannoni et al. 2020) and, for Titan, the time-variable field as well (Iess et al. 2012). The Doppler tracking data that will be obtained close to Europa will allow the determination of key static and time-variable gravity field parameters over the course of the entire Europa Clipper mission. Simulations conducted with the expected Doppler observation accuracy show that the tidal Love number k_2_ can be recovered to an accuracy of < 0.05. For more details, see Mazarico et al. (2023).

A better constraint on Europa’s interior and in particular on the ice shell thickness and rheology can be obtained if $k_{2}$ is combined with the tidal Love number $h_{2}$, which is an expression of the radial tidal deformation. Measuring both tidal Love numbers comes with the advantage of mitigating some of the ambiguities that arise from only one Love number alone (Wahr et al. 2006; Park et al. 2015).

The Europa Clipper mission will use the VHF component of the radar sounder REASON as an altimeter. The VHF antenna has a wavelength of 5 m and an inherent range resolution of 15 m in free space. During the tour, the spacecraft groundtracks over the surface of Europa intersect, leading to “cross-over” points. If the individual flybys occur at different positions of Europa in its orbit around Jupiter, i.e., at different true anomalies, then the position of the surface at these intersections will have shifted between groundtracks. In the presence of a subsurface ocean, the expected radial tidal deformation is on the order of 30 m, predominantly dependent on ice shell thickness. Since a radar sounder is illuminating a large area of the surface (on the order of several to tens of km^2^), the radar returns have some ambiguity in the presence of surface topography. To mitigate this uncertainty and actually exploit the multiple returns from different locations, stereo imaging of the cross-over points is needed. This knowledge of the topography will allow Europa Clipper to perform multiple altimetry measurements simultaneously and, assuming that these are statistically independent, to increase the measurement accuracy below that of the inherent range resolution (Steinbrügge et al. 2018). The $h_{2}$ measurement will therefore be a combined measurement between REASON and EIS). Simulations accounting for the tour geometry and the radar performance estimate the accuracy to which h_2_ can be recovered to about 0.1, but this estimate depends strongly on the surface roughness (Steinbrügge et al. 2018).

### Auxiliary Measurements

#### Static Gravity

In addition to the time-varying gravity field (i.e., tides), the Gravity and Radio Science investigation will measure the static components of the long-wavelength, global potential field (primarily spherical harmonic degree 2). Because of the rapidly varying spacecraft altitude, regional to local gravity anomalies are better constrained by line-of-sight residuals (e.g., James 2016). Doppler shifts of the radio signal during individual flybys arise from along-sight accelerations caused by mass anomalies on and below the surface. After known sources of acceleration are removed, the remaining line-of-sight residuals can be tied to these gravity anomalies. Mass anomalies hundreds to thousands of km across on Ganymede were detected during relatively distant (hundreds to thousands of km) flybys by the Galileo spacecraft (Anderson et al. 2004; Palguta et al. 2006, 2009); Europa Clipper’s flybys will be closer, with commensurately finer resolution.

Localized gravity anomalies will arise from internal mass density anomalies and topography at the interfaces between layers with different densities. Because of Europa’s compositional structure, the largest density contrast exists at the rock–water interface, though distance will attenuate the shortest scale component of that signal (e.g., Pauer et al. 2010; Dombard and Sessa 2019). The surface also represents a strong density contrast, though this signal could in principle be removed via gravity predicted from corresponding topographic profiles measured by Europa Clipper, perhaps revealing the near-surface density of the ice shell. Beyond that, mass density anomalies such as frozen or liquid brine pockets within the ice shell might be detected; such anomalies may be compared against other Europa Clipper data (e.g., radar, geology from imagery, surface composition) for interpretation.

If Europa behaves like a static fluid, i.e., is hydrostatic, then its shape or gravity can be used to determine its MoI directly via the Darwin-Radau relationship, which relates the MoI of a hydrostatic body to its rotational speed and shape. An important goal of the Gravity and Radio Science investigation is to measure the low-degree (particularly degree-2) gravity moments to determine how close Europa is to hydrostatic equilibrium. Most approaches to date have used the hydrostatic assumption to derive Europa’s MoI from the estimated C_22_ coefficient and assuming a ratio of J_2_/C_22_ equal to 10/3 (e.g., Schubert et al. 2009). Gomez Casajus et al. (2021) recently re-analyzed the Galileo data and retrieved coefficients C_22_ and J_2_ independently, finding that these are compatible with a body in hydrostatic equilibrium within an uncertainty of 1-$\sigma $. Precise independent estimates of the two main coefficients will tell us with more rigor whether Europa is hydrostatic (Tricarico 2014).

Cold silicate objects like Vesta and the Moon show pronounced departures from hydrostatic equilibrium, while icy bodies like Titan or Enceladus depart slightly from this state. For the latter two bodies, correlations between the gravity and surface topography have been used to infer not only the moment of inertia, but also the degree of compensation (and hence the thickness) of the ice shell (Hemingway et al. 2013; Iess et al. 2014). It may be possible to apply this approach at Europa, but it is also possible that non-hydrostatic components arise from the silicate interior (e.g., Dombard and Sessa 2019). In this case, one would not expect any correlation between the surface topography and gravity, and thus the Titan/Enceladus approach cannot be used. Nonetheless, in such a case, one might be able to draw inferences about whether Europa’s silicate interior was relatively cool and rigid with limited tidal dissipation or whether there are low-degree signals arising from convection within the silicate mantle. The accumulation of flyby Doppler data should allow determination of Europa’s static gravity field up to at least degree 5. More detail on the Europa Clipper Gravity and Radio Science investigation can be found in Mazarico et al. (2023).

#### Geodesy

Geodetic observations provide critical constraints to characterize and understand Europa’s interior structure, including its radial mass distribution and ice shell properties. Fundamental properties like shape, gravity, and rotation state will be assessed by the Europa Clipper mission and will provide the frame for many investigations. Many of these observations will be multi-instrument investigations, as the global shape model will be derived from visible and ultraviolet imaging (EIS, Europa-UVS) and radar altimetry (REASON) data. The geodetic information that is of particular interest for interior science includes tidal deformation, shape-determination, librations, obliquity, as well as local gravity anomalies. The tidal response of Europa has been addressed in the previous section. The remaining observations are briefly described here.

The shape of Europa provides insight into the structure and thickness of its ice shell (e.g., Nimmo et al. 2007) and the distribution of tidal heating (e.g., Ojakangas and Stevenson 1989). Europa Clipper will determine Europa’s shape through a combination of REASON altimetry profiles, EIS limb profiles, and Europa-UVS occultation measurements (Abrahams et al. 2021). REASON altimetry profiles have an accuracy on order of 10 m (Steinbrügge et al. 2018). Due to the flyby nature of Europa Clipper trajectory, REASON altimetry measurements are concentrated near Europa’s sub- and anti-Jovian hemisphere, which limits the spherical harmonic degree to which Europa’s shape can be determined. EIS limb images at spatial scales as low as ≤1 km/pixel will be acquired by the EIS NAC and WAC cameras. Limb profiles can be derived from these images with a precision of ∼0.1 pixels (Thomas et al. 2007), resulting in shape information with an accuracy on the order of 100 m. Although the EIS profiles are an order of magnitude lower in resolution compared to the REASON altimetry profiles, they are more spatially extensive. Abrahams et al. (2021) has shown that these gaps can be filled by Europa-UVS stellar occultation measurements, substantially increasing the achievable spherical harmonic degree – from 8 to 13 for very conservative assumptions about REASON performance and without limb profiles. The UVS measurements have timing accuracy of as low as 1 ms, depending on the instrument operating mode and desired precision, which for Europa-Spacecraft relative velocities of ∼1 km/s equate to measurements of chords across the body of Europa with a precision of ∼1 m (Abrahams et al. 2021).

The amplitude and period of Europa’s libration potentially provides information about the thickness and rigidity of the ice shell. On average, Europa presents the same face toward Jupiter, but the eccentricity of its orbit leads to a variable orbital velocity, and hence libration. The forced libration, which is the component due to Jupiter’s gravitational torque on the dynamic figure of Europa, is of interest in potentially providing geophysical information. However, while the libration amplitude can be large (130 m; Bills 2005), the sensitivity to the ice shell thickness is poor except in the case of a very thin crust (Van Hoolst et al. 2013).

The EIS NAC will acquire a geodesy dataset of 50–100 m/pixel framing images, covering all longitudes and obtained as much as possible near the extrema of libration. Nevertheless, achieving the required measurement precision of between 5–10 m would be challenging for the mission. Therefore, determination of the libration is of less interest than other methods for determining ice shell thickness. A somewhat coarser measurement (better than 50 m precision) in principle might be sufficient to determine whether the crust is decoupled from the silicate mantle (Verma and Margot 2018). However, since the solid Europa libration amplitude is ∼135 m, right in the middle of the predicted values for a Europa with an ocean and ice shell, even here a libration measurement appears unfortunately not to be diagnostic.

**Fig. 5:**
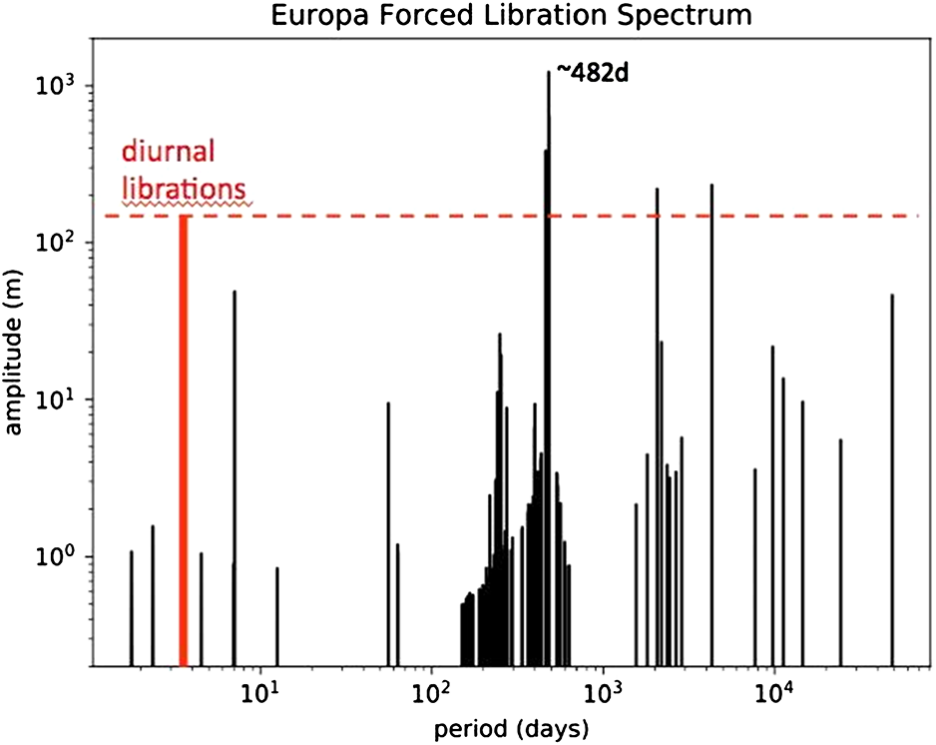
Spectrum of the forced libration of Europa. Adapted from Rambaux et al. (2011)

Longer-period librations (>10 days) driven by the Laplace resonance can be even larger; the maximum amplitude is over 1 km at a 482-day period (Rambaux et al. 2011; Fig. 5). Although these forced librations are not sensitive to the properties of the interior structure, it is essential that they be tracked so that surface features can be accurately located. Radar ranging crossovers can be used to improve the accuracy of the spacecraft position and orbit, but the crossover points need to be co-registered with the imaging data.

The radial mass distribution of a planetary interior can be estimated from measurements of the MoI. However, non-hydrostatic components of Europa’s gravity field arising from the mass distribution in the deep interior may complicate the determination of the MoI. An alternative approach to retrieve Europa’s internal mass distribution involves the accurate estimation of the pole obliquity, $\epsilon $, which is the angle between a body’s spin axis and orbit-plane normal (Bills and Nimmo 2008). This procedure assumes that the body is in a Cassini state, a state of minimum energy that accounts for the coplanarity of spin, orbit precession, and orbit angular velocity (Peale et al. 2002). By combining measurements of Europa’s ephemeris and orientation, Europa Clipper will allow us to confirm whether the icy moon is in a Cassini state, and thus whether we can measure the polar moment of inertia directly from the measured obliquity, which is expected to be ∼0.05° (Chen et al. 2014). Radiometric Doppler data collected by Europa Clipper during all close encounters will provide critical information on Europa’s ephemerides. Doppler data acquired near the pericenter are very sensitive to the relative position of the spacecraft with respect to the moon. Gravitational torques exerted on the ice shell by an asymmetric silicate interior should provide a strong coupling mechanism on the long timescales of the pole precession (Van Hoolst et al. 2008). Visible imaging (discussed in the REASON investigation publication in this collection) and gravity science data (Mazarico et al. 2023) are then expected to provide consistent measurements of Europa’s obliquity with these independent investigations.

One of the key factors affecting the Jovian system’s dynamics is the tidal interaction between Jupiter and the Galilean moons (Ojakangas and Stevenson 1986; Hussmann and Spohn 2004). The orbital energy dissipation due to the tides that Europa, Io, and Ganymede raise on Jupiter significantly perturbs the orbital semi-major axis of these moons which may lead to substantial changes over the long term. There is no consensus on the contribution of this dissipation mechanism to the dynamics of the Galilean moons, as current results disagree both in order of magnitude and sign (Lainey et al. 2009; Jacobson 2015). That is, not only is the scope of the tidal interaction uncertain, but even whether the moons are accelerating (i.e., moving toward Jupiter) or decelerating (i.e., drifting away from it) and moving in or out of resonance. It is therefore equally unclear if Europa is in equilibrium. The oscillation of Europa’s eccentricity can take place on the order of 100 million years which would lead to episodical variations in the intensity of tidal heating for all satellites bound in the Laplace resonance. In this scenario, the ice shell thickness of Europa would vary between 3 and 70 km (Hussmann and Spohn 2004), and phases of quasi-steady evolution would alternate with phases of heating and cooling, potentially even leading to resurfacing events as has been suggested for Ganymede’s past (Showman and Malhotra 1997). To address the question of equilibrium the amount of tidal dissipation must be measured as well as the change in semi-major axis (see e.g., de Kleer et al. 2019). Alternatively, using astrometric observations of the Europa Clipper mission in concert with JUICE observations, the stability of the Laplace resonance could be verified by a measurement of the migration timescale of all three satellites within the Laplace resonance (Dirkx et al. 2017).

#### Surface Heat Flux

Because of Europa’s young surface age, geological activity is expected and some forms of activity (e.g., rifting, subsurface intrusions, plume eruptions) may lead to surface thermal anomalies. Double ridges in particular are promising locations for thermal anomalies (Nimmo et al. 2002; Dombard et al. 2013). The detectability such activity depends on the area of the anomaly, the anomaly lifetime, and the mechanism for the activity. Preliminary analysis suggests that surface eruptions could be detectable for a few hundred years after the eruption, while concentrated subsurface heat sources like the Enceladus tiger stripes (Spencer et al. 2006; Howett et al. 2011) would be readily detectable (Hayne et al. 2017).

In contrast to the well characterized plume at the south pole of Enceladus (Howett et al. 2011; Postberg et al. 2018), the extent to which plume activity on Europa will be accompanied by thermal anomalies is still unclear (Rathbun and Spencer 2020). Nevertheless, given the stress conditions within the ice shell, it is likely that any plume material on Europa originates in discrete fluid pockets perched at shallow levels in the ice shell, rather than directly from the ocean ((Fagents et al. 2000; Fagents 2003; Manga and Wang 2007)). The heat given off by such a reservoir would produce detectable thermal anomalies as long as the source depth was <1 km (Hayne et al. 2017). Recent modeling suggests that liquid reservoirs perched in Europa’s ice shell could undergo cooling for at least tens of thousands of years before completely freezing (Quick et al. 2021, 2022). Even if not currently active or associated with a detectable surface thermal anomaly, recent plume activity might be identified by fresh plume deposits (Quick and Hedman 2020). Plume fallout may have distinct thermal properties at the surface: plume deposits might be composed of smaller ice particles than the typical Europa surficial material and may result in detectably lower thermal inertia units.

#### Topographic Constraints on Ice Shell Thickness

Constraining Europa’s average ice shell thickness is one of Europa Clipper’s baseline science objectives; however, detecting regional variations in its thickness is also of high science value. Material exchange between Europa’s surface and ocean is critical for maintaining a habitable ocean environment, and such exchange may occur more easily in thinned portions of the ice shell. Ice shell thinning may also reveal the existence of water perched in the ice shell (Schmidt et al. 2011), and constrain, at broader scales, thermal structure and heat transport mechanisms (e.g., Nimmo et al. 2007). REASON sounding measurements are the principal method for constraining ice shell thickness; however, the surface topography itself provides additional constraints, especially in the limit of a thick shell. In the absence of elastic stresses (true over regional length scales), isostatic adjustment of the ice shell will result in thinner portions of the shell lying topographically lower than thicker portions. Topographic stresses should drive flow which removes ice–shell variations over time, so the existence of thickness variations implies on-going geologic processes and/or a very thin shell (Stevenson 2000). Current observations of Europa’s long-wavelength topography suggest that the ice shell has relatively uniform thickness, implying that either the shell thickness variations are not detectable with current observations (and the ice shell is relatively thin) or that the ice shell is indeed uniform, and lateral flow is efficient or the ice shell is convecting (Nimmo et al. 2007). Topography measurements by Europa Clipper can distinguish between these possibilities by refining our knowledge of Europa’s regional-scale topography. The principal method of acquiring such topography is REASON altimeter, EIS WAC three-channel stereo (NAC targeted stereo is also possible), and limb profiles. All of these datasets must be controlled to each other and co-analyzed for the best results.

The measurement of transects across topographic elevations either from digital terrain models or by the collection of REASON altimetry groundtracks or limb profiles allows determination of the thickness of the elastic portion of the ice shell. On terrestrial as well as on icy bodies, the topography at short baselines is often supported by the flexure of the cold upper layer of the crust. By measuring this flexure from the elevation profiles and by assuming a range for the rheological properties of the ice, the effective elastic thickness of the shell can be inferred. This technique has been applied to Europa and Ganymede based on Galileo images (e.g., Williams and Greeley 1998; Nimmo et al. 2002, 2003a). Current best estimates for the elastic thickness of Europa range from 0.2–3 km as measured from the blocks inside Conamara chaos (Williams and Greeley 1998) and a few hundred meters from double ridges (Billings and Kattenhorn 2005), up to 4–11 km as inferred from a prominent plateau southwest of Cilix crater (Nimmo et al. 2003a). Since the elastic strength of the ice depends on the temperature structure, which itself depends on the entire ice shell, additional assumptions about the thermal state further lead to constraints on the ice shell thickness itself (Nimmo et al. 2003a).

#### Surface Composition

MISE, EIS, and Europa-UVS will map Europa’s surface composition at global and regional scales at unprecedented resolution (> 70% of the surface at 10-km/pixel scale) over a 0.1–5 μm spectral range. EIS color data can reveal color centers characteristic of irradiated chlorides. Oceanic material that erupted onto the surface (e.g., at chaos regions or condensed as cryovolcanic plume fallout) will be sputtered by high-energy particles and then sampled by MASPEX and SUDA. Together with Europa-UVS, these instruments will measure the abundance of key exospheric species (e.g., H_2_O, O_2_, H_2_) and trace organics and salt derivatives (Na and K). MASPEX measurements of the composition of low-weight organic volatiles as well as potential ice grains may point to the presence of clathrate hydrates in the crust (see Becker et al. this collection). These observations will support various interior investigations, in particular that of ECM. Compositional data on endogenic salts and H_2_/CO_2_/H_2_O ratios in plumes would constrain the composition of suboceanic rocks (Becker et al. this collection) and yield indirect constraints on Europa’s density profile. EIS and E-THEMIS bring geological context in order to infer the processes involved in the emplacement of surface salts and organics and distinguish between compounds of endogenic vs. exogenic origin (such as implanted sulfur from Io vs. oceanic sulfates) (see Becker et al. this collection). MASPEX, Europa-UVS, PIMS, and SUDA measurements will also provide a better understanding of the particles entering the exospheric region from other (most likely Io) sources.

## Synthesis

### Synergy

In a broad sense, there are three unknown interior quantities that the Europa Clipper mission will determine: the ice shell thickness, ocean thickness, and ocean salinity, and three primary investigations to address them: magnetic induction, subsurface sounding, and tidal deformation. The electromagnetic induction response of Europa’s ocean takes the form of an induced magnetic field, which can be expressed in terms of an amplitude (often called the normalized amplitude) and a phase delay. This response depends on three parameters: the depth to the ocean, the electrical conductivity of the ocean, and the ocean thickness. In order to obtain all three ocean parameters, the response of the ocean at three widely separated frequencies is required. Indeed, nature does provide strong signals at three frequencies, the synodic rotation period of Jupiter (11.2 h, signal strength ∼200 nT), the second harmonic of the synodic rotation period (5.5 h, signal strength ∼15 nT) and the orbital period of Europa (85.2 h, signal strength ∼15 nT) that can be used to determine the properties of the ocean.

**Fig. 6:**
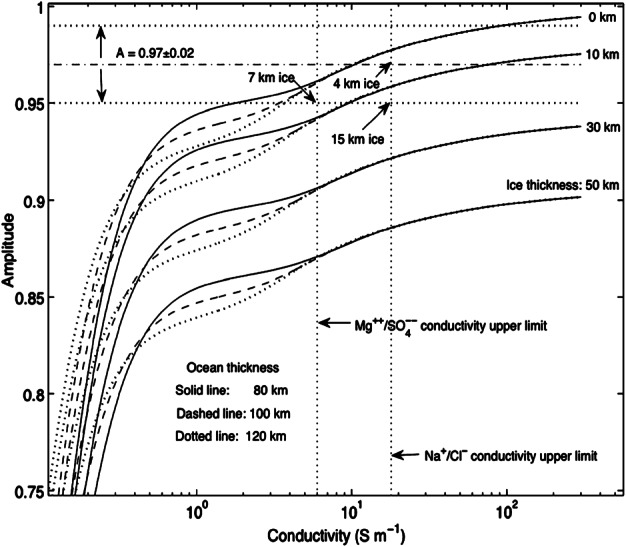
The amplitude response as a function of ocean conductivity, ocean thickness, and ice shell thickness for a three-layer model. The range of response factor deduced by Schilling et al. (2004) are marked by horizontal dotted lines. The upper limit imposed on the conductivity of the solution from saturation effects are marked by the two vertical lines. Figure reproduced from Hand and Chyba (2007)

**Fig. 7:**
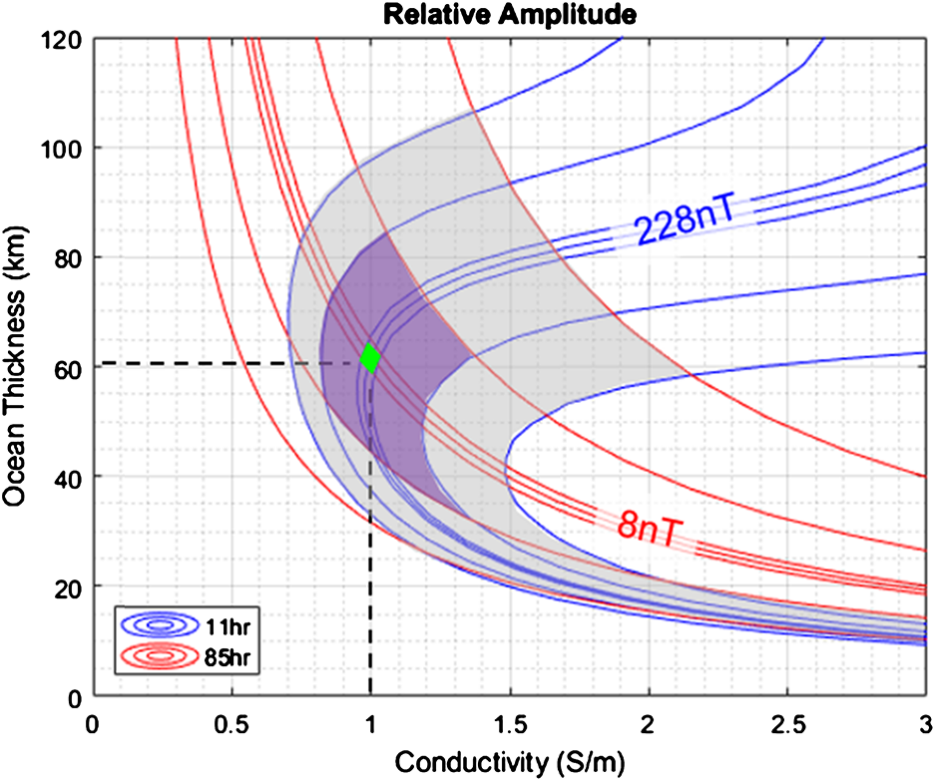
The dipolar surface induction field created by the interaction of Europa with Jupiter’s varying field at the two principal frequencies (T = 11.1 h and T = 85.2 h) for a range of conductivities and ocean shell thicknesses.

The normalized amplitude (see Fig. 6) varies strongly as a function of all three parameters. In general, ocean thickness only modestly affects the amplitude of the response at the synodic frequencies. However, the depth of the ocean below the surface and the conductivity of the ocean strongly affect the amplitude response. For ocean conductivities >∼1.0 S/m, the amplitude response, which is dipolar, approaches unity at the surface of the ocean and drops off with the cube of distance outwards towards the surface. Figure 7 shows the amplitude response of Europa at two of these frequencies (the synodic and orbital period frequencies) as a function of ocean conductivity and thickness. In certain optimum conditions (large thickness and high conductivity), both of these parameters can be obtained uniquely from these responses, if the amplitudes can be determined with a precision of 1.5 nT or better. In order to constrain ocean parameters for all conditions, further constraints on one of the parameters from other experiments is desired. For example, if the ice is thin and that thickness can be accurately measured from the ice penetrating radar measurements, Fig. 7 shows that the ocean conductivity can then be inferred quite accurately (further improvement is possible with the 5.5 h signal, but this is much lower amplitude than the full synodic period signal). If, on the other hand, the ocean conductivity can be obtained independently from mass spectrometry of the salt components of an ocean plume, very accurate estimates of the ice thickness can be obtained from Fig. 7.

In an idealized case, these three investigations alone would provide us with a solution to the three quantities identified in the Europa Clipper’s Level-1 science requirements. However, such precision would require both that the measurements themselves have zero uncertainty (which is an unrealistic scenario) and that the equations are linearly independent. Additional information to resolve this potential inherent nonlinearity is provided in the form of the auxiliary measurements described in Sect. 2.4. These additional datasets can provide context, which can serve to narrow the parameter space and simplify the relationships.

**Fig. 8:**
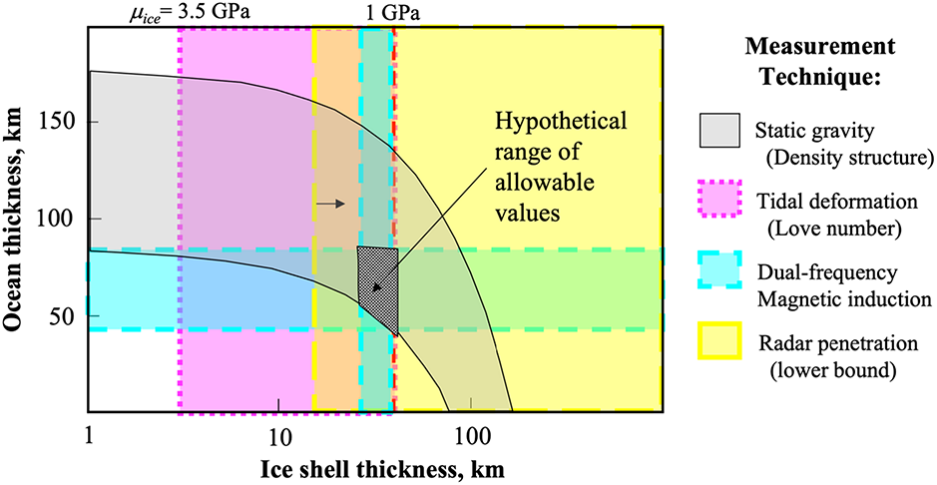
Overlapping measurements combine to constrain the ice shell and ocean thickness. In the example illustrated here, the “true” mean ice shell thickness is 20 km, the upper 6 km of which is rigid. The subsurface ocean is 60 kim thick and has a conductivity of 1 S/m. See text for additional details.

Although no single instrument can fully characterize the ice shell, a *combination* of measurements provides a much more complete picture. Figure 8 provides a hypothetical example of how this synergy can work in practice. In this example, the “true” ice shell thickness (global averages) is assumed to be 20 km, with a 6 km rigid lid sitting above a convecting interior, while the ocean beneath is 60 km thick and has a conductivity of 1 S/m.

Static gravity coefficients constrain the moment of inertia (especially when hydrostatic equilibrium pertains) and thus the combined thickness of the ice shell plus ocean (Schubert et al. 2009). The uncertainties in this tradeoff (grey swath) arise mainly from uncertainties about the density of the core and mantle.

The tidal response of $k_{2}=0.245$ can be used to infer a rigid shell thickness, but only if the shell rigidity is known. The real rigidity of the shell depends on the viscosity and porosity structure, so here a range of 1–3.5 GPa is considered (red box). Uncertainties in the ice shell or ocean density will contribute further uncertainty, but can be mitigated to some extent if $h_{2}$ can also be measured (Wahr et al. 2006). The actual measurement uncertainties in $k_{2}$ are insignificant compared to these other sources of uncertainty.

In the particular hypothetical model of Europa depicted in Fig. 8, radar sounding will not penetrate to the base of the ice shell because of the strong attenuation of radio waves in the warm ice in the lower shell. However, it will penetrate close to the base of the cold, rigid lid, providing a lower bound on the shell thickness of 6 km (yellow box).

Finally, for the particular parameters assumed here, the mutli-frequency magnetic sounding technique will allow the conductivity, ocean thickness, and total shell thickness to be determined independently. Assuming a measurement uncertainty of ±1.5 nT, the error bars are ±30 km for the ocean and ±5 km for the shell (blue boxes). Unlike the $k_{2}$ or static gravity techniques, here the measurement errors dominate the uncertainty in the derived parameter values.

In this particular example, it is the combination of static gravity and magnetometry that together provide the main constraints on the shell and ocean thickness, giving combined uncertainties of ±5 km and ±10 km, respectively. But in other hypothetical examples, other techniques will become important. For instance, if the ice shell is thin (<10 km) then the magnetometer uncertainties will prevent a useful shell thickness estimation, but in this case the radar may be more likely to penetrate to the base of the shell and provide a direct measurement. Multiple measurements also provide some redundancy in case of unanticipated instrument failure.

Auxiliary measurements, not shown in Fig. 8, may also prove useful. For instance, a thin shell would result in a high heat flux, potentially detectable by E-THEMIS. Conversely, in the case of a thick, rigid shell, gravity and topography measurements might be able to determine the thickness of the rigid, upper shell.

Although more challenging, multiple measurement techniques may also provide insight into the deeper interior. Local magnetic anomalies would be evidence for the existence of a core, while departures from hydrostatic equilibrium and/or large gravity anomalies could be indications of a cold, rigid interior. Conversely, a shell only a few km thick would imply a highly deformable interior; this would also give rise to a significant lag in the $k_{2}$ tidal response.

While multiple investigations directly constrain the ice shell thickness, they address different aspects of it. As an example, magnetic induction looks at the depth to the ocean, which depends on the total ice shell thickness. Subsurface sounding could provide this measurement if unambiguous detection of the ice–ocean interface was possible, which is however unlikely (Blankenship et al. 2009). Instead, it is likely to be more sensitive to the thickness of the conductive layer. Tidal deformation is sensitive to a combination of the total ice thickness and the rheology; or in some sense the thickness of the elastic portion of the ice shell. These different aspects of the shell thickness can be reconciled using auxiliary data. The long-wavelength static gravity measurements will tell us if the ice shell is in hydrostatic equilibrium, placing a minimum bound on the ocean thickness. Surface heat flux can be used to estimate the thickness of the conducting portion of the ice shell. The elastic thickness can be independently estimated using along-track topography profiles. In this way, much more detailed information is gathered about the structure of the ice shell than its overall thickness. In the following sections, we explore the geological, geophysical, and geochemical context that will be used to place constraints on the interior structure of Europa.

### Geology and Geophysics

The average surface age of Europa’s icy shell is estimated to be between 40 and 90 Myr (Bierhaus et al. 2009). Thus, the surface of Europa is one of the youngest in the solar system, and indicates recent or current geologic activity that erases or replaces older terrains through resurfacing processes. The terrains associated with resurfacing are fully discussed in Daubar et al. (this collection), and can roughly be broken up into tectonic processes and the processes that result in the formation of chaotic terrains. Because these landforms are the surface expression of processes occurring deep within the ice shell, their characterization and associated observations can reveal information about the interior state, structure, and evolution of the ice shell and ocean.

Europa’s surface deforms along convergent (e.g., Kattenhorn and Prockter 2014), strike-slip (e.g., Nimmo and Gaidos 2002), and extensional (e.g., Prockter et al. 2002; Howell and Pappalardo 2018) tectonic boundaries between semi-rigid plates that have been likened to plate tectonic processes on Earth, except that extension appears to dominate far more on Europa than on Earth (Prockter et al. 2002; Kattenhorn and Prockter 2014). Additionally, Europa’s surface is heavily modified by the formation of chaotic terrains, which comprise regions of tilted and rotated blocks of pre-existing terrains in a fine hummocky matrix of ice (e.g., Greenberg et al. 1999). The interior processes associated with the formation of chaos terrain may involve hot diapirism of solid ice, in situ melting, and/or brine injection from the subsurface ocean (e.g., Collins and Nimmo 2009; Head and Pappalardo 1999; Schmidt et al. 2011).

The surface expression of interior deformation is controlled by the thermal and mechanical state of the ice shell, which in turn depends on the composition and heat budget of the deeper interior. The global heat budget is largely driven by radioisotope decay within the silicate mantle and the dissipation of gravitational tidal energy as heat within the ice shell, ocean, and mantle. Landform geology can be characterized through geologic mapping, morphological characterization of DEMs, and heat flow analyses (Daubar et al. this collection), with additional context provided by compositional mapping (Becker et al. this collection), These analyses provide constraints on the ranges of plausible brittle and elastic behaviors, thermal and mechanical properties of the ice shell, and the geodynamic state of the interior. Landform geology can therefore help to elucidate the sources of energy that support resurfacing on Europa to maintain its young average surface age.

The geologic processes occurring within Europa’s icy shell, ocean, and rocky interior facilitate the transport of materials and energy throughout the body, and are key to establishing and maintaining chemical disequilibria (Hand et al. 2007). Thus, geologic transport processes within Europa’s interior directly affect habitability and the potential for life to emerge and persist (Vance et al. this collection). Within the icy shell, tectonism, convection, diapirism, cryovolcanism, and impacts each may contribute to material transport. Thermal convection within the ice shell may be driven by both the temperature differential between the warm subsurface ocean (e.g., McKinnon 1999) and cold surface and the volumetric generation of heat associated with dissipated tidal energy (e.g., Vilella et al. 2020), with salts potentially playing a role as well, in the form of compositional buoyancy (e.g., Pappalardo and Barr 2004). Europa is likely in a stagnant lid regime, with a crust that is not experiencing continuous overturn (Howell 2021). Thus, convection is expected to play a primary role in transporting material between the ocean and the base of the conductive lid, and laterally across the length scale of convective cells, on timescales of 10^4^–10^5^ yr (Showman and Han 2004). Additionally, thickness gradients in the ice shell may cause ductile and convecting ice to participate in meridional flow, potentially transporting materials over great lateral distances (Ashkenazy et al. 2018).

Extensional tectonics associated with band formation may locally thin the mechanical lithosphere of the icy shell, increasing the surface heat flow locally (Daubar et al. this collection), and permitting the surface exposure of warm ice from deep within the ice shell (Howell and Pappalardo 2018). Ice recently frozen into the ice shell from the subsurface ocean may be transported to the surface, and exposed on timescales of <1 Myr (Howell and Pappalardo 2018). Convergent processes on Europa are relatively rare, and/or difficult to locate in Voyager and Galileo observations of Europa (Sarid et al. 2002). These processes may be associated with cold thermal anomalies resulting from the local deepening of cold isotherms and corresponding reduction in surface heat flow. Convergence associated with the “subsumption” of warm ice – defined as the downward penetration and resorption of icy slabs into the ice shell – may similarly be critical for surface–to–ocean material exchange (Kattenhorn and Prockter 2014). While observations of convergent processes on Europa are rare, Culha et al. (2014) showed that some compression is being accommodated at double ridges. Sustained subsumption could permit the downward transport of surface material to the subsurface ocean on timescales of 10^6^–10^7^ yr (Kattenhorn and Prockter 2014).

The possibility of direct vertical transport of subsurface ocean water into the ice shell through sill injection has been explored in depth for Europa (e.g., Manga and Wang 2007; Michaut and Manga 2014). However, this process is controversial due to mechanical arguments related to the viscous relaxation of cracks and difficulty in generating the stresses required for sill injection (e.g., Collins and Nimmo 2009; Michaut and Manga 2014; Craft et al. 2016). Water that is injected into the elastic portion of the ice shell at depth, and potentially water formed by in situ melting, may create an overpressure when recrystallizing and cause vertical transport (e.g., Schmidt et al. 2011) and surface eruption of subsurface material (e.g., Lesage et al. 2020). In the absence of overpressure, exsolution of volatiles (Crawford and Stevenson 1988) may be able to drive eruptions. Alternatively, in situ melting beneath frictionally heated tectonic boundaries or chaos regions may result in dense water filled pores that sink rapidly to the ocean in discrete “porosity waves” (Kalousová et al. 2014).

The subsurface ocean may also play a major role in the global transport of materials within Europa’s interior, influencing the ice shell composition and state at the ice–ocean interface (e.g., Vance et al. 2019), and potentially participating in hydrothermal activity and redox reactions at the seafloor (e.g., Vance et al. 2007). Due to a moderate rotational influence, convection in Europa’s ocean is thought to produce few zonal jets and Hadley-like overturning circulations with a maximum heat flux near the equator (Soderlund 2019). This regime may help drive geologic activity via thermo-compositional diapirism in the ice shell (Soderlund et al. 2014). In contrast, electromagnetic pumping may lead to a single westward jet that may contribute to non-synchronous rotation and ohmic dissipation that is concentrated in a thin layer near the ice–ocean interface near the poles (Gissinger and Petitdemange 2019). Additional flows will be driven by tidal forcing and libration of the ice shell, some of which will focus enhanced currents along internal shear layers in the ocean (e.g., Rovira-Navarro et al. 2019; Hay et al. 2021). Enhanced vertical mixing is likely to occur along these layers, although heat generation through viscous dissipation does not seem to be a significant contributor. Vertical transport and mixing in the ocean may also be influenced by the salinity. Fresh water near the melting point has a negative coefficient of thermal expansion. However, if the ocean salinity exceeds 2.2%, the thermal expansion coefficient may turn positive. In this scenario, hydrothermal vents could generate buoyant plumes of water, which would drive convection in a thick ocean layer below a colder, stratified layer described above (Kang 2022; Kang et al. 2022; Bire et al. 2023). Europa Clipper measurements will constrain ocean flows and test these hypotheses as described in Sect. 1.2.3.

The mechanical properties and thermal state of the ice shell may be inferred by a combination of geological and geophysical measurements. A preliminary essential assessment concerns the total ice shell thickness, which can be retrieved by combining magnetic induction (Sects. 2.1, 3.1), radar sounding (Sects. 2.2, 3.1), tidal monitoring (Sects. 2.3, 3.1), and geodesy (Sect. 2.4) techniques. Once the average thickness is constrained, inversion of static gravity and long-wavelength topography data will allow the determination of lateral thickness variations, using an approach that has been successfully applied to Saturn’s moons using the Cassini data (e.g., Nimmo and Bills 2010; Nimmo et al. 2011; Lefevre et al. 2014; Beuthe et al. 2016; Hemingway et al. 2018; Čadek et al. 2019). Lateral variations in the ice shell thickness will be a key diagnostic to determine whether Europa’s ice shell is currently in a convective or conductive state (Nimmo et al. 2007), to understand the global stress balance (e.g., Howell and Pappalardo 2019), and to assess potential heat flux anomalies from the ocean. This approach has previously been applied to Titan and Enceladus, based on Cassini data (Kvorka et al. 2018; Čadek et al. 2019). Interpretation of magnetic sounding measurements from close flybys may also be able to provide constraints on induced quadrupole moments, which may then be used to constrain the shape of the conducting ocean layer (Styczinski and Harnett 2021). If marked lateral variations in ice shell thickness are in fact present, several investigations are likely to provide converging lines of evidence to support their detection. The rheology of the silicate mantle is not well known, and the mantle cannot be as easily observed as the ice shell. However, if the silicate mantle is as dissipative as Io, tidal heating in the large silicate portion of the body may be substantial. Within the rocky interior, tidally induced magmatism may continue episodically to the present day, transporting volatiles from the rocky interior to the seafloor through volcanism (Běhounková et al. 2021).

Local and regional topography can be combined with morphological interpretation to provide constraints on the near surface mechanical (elastic/brittle) properties, and indirectly on the near-surface thermal gradients. Such a technique has already been applied to the Galileo data. However, detailed topography data is available only for a limited set of sites (e.g., Nimmo et al. 2003a; Billings and Kattenhorn 2005), and complementary information on the near-surface ice properties is not available. Radar sounding will constrain near-surface porosity (Sect. 2.2) and potentially the depth of porosity closure (Nimmo et al. 2003b) and thickness of the conductive lid (Kalousová et al. 2017), which will be essential to reconstruct the thermal profile and its lateral variations. Detection of potential subsurface liquid reservoirs from radar sounding (Sect. 2.2) and anomalous thermal emission anomalies (Sect. 2.4.3) may also reveal local upwellings and heat source anomalies, which will be essential to assess the level of present-day thermal activity of the ice shell and efficiency of exchange with the subsurface ocean. Large, saline reservoirs may also have an influence on magnetic induction signals (Sect. 2.1), especially for the closest flybys. Magnetic induction may provide an additional line of evidence to support the detection of such reservoirs.

Long-wavelength topography and gravity can be used to constrain the lateral variations in shell thickness, providing the thermal state and global dynamics of the ice shell. In combination with heat production within the ice shell that is expected to vary laterally (Ojakangas and Stevenson 1989; Tobie et al. 2003), strong heat flux anomalies coming from the seafloor and heat flux patterns due to oceanic circulation and tidal dissipation can lead to a modulation of the ice–ocean interface. Local and regional thinning of the ice shell may reveal active seafloor hotspots or at least an anomaly in the global oceanic circulation, which may have various origins (e.g., Hay and Matsuyama 2019; Soderlund et al. 2020). Local ice shell thinning combines with gravity anomalies and detection of local enhancement in H_2_, CH_4_, and other volatile gases may confirm the existence of ongoing seafloor volcanic activity (Běhounková et al. 2021).

### Surface Material Emplacement

Material on the surface of Europa is believed to have been emplaced in a variety of ways, such as intrusions through fractures, cryovolcanism, diapirism, and deposition of plume material. Furthermore, surface material may have been modified since exposure. Space weathering may alter the nature of salt compounds on a surficial scale (millimeters to a few centimeters). On the other hand, gardening by micrometeorites locally exposes fresh material on a similar scale, which can help entangle surficial effects and reveal the true nature of material sourced from the deep interior. The composition of most materials should be preserved in the ejecta blanket, except maybe for fragile material like organics (e.g., Bowling et al. 2020).

Recent models leveraging knowledge developed for terrestrial sea ice suggest various mechanisms for introducing salts into Europa’s ice shell. Buffo et al. (2020) have explored the dependence of salt trapping in the crust as a function of thermal gradients. Wolfenbarger et al. (2022a) expand on the work of Buffo et al. (2020) to examine how different mechanisms of ice accretion can influence salt entrainment at the low temperature gradients expected at the ice–ocean interface. Salts may be transported further into the ice shell (e.g., via convective plumes) and concentrated in the shallow subsurface. The salts may evolve during their journey toward the surface, and this evolution is not well understood (see Vance et al. 2021b). For example, the salts may interact with clathrate hydrates (e.g., Méndez et al. 2017) and evolve under reheating from tidal energy (e.g., Muñoz-Iglesias et al. 2019) or other sources. In general, the chemical evolution of salts trapped in the shell is an area of active investigation, as elaborated by Vance et al. (2021b). Studies of chemical fractionation in both natural and artificial ice have shed some light on processes that may alter the chemistry of oceanic material entrained through freezing and have been used to hypothesize the enrichment and depletion of certain impurities in an ice shell (Wolfenbarger et al. 2022a).

Salts trapped in the shell may contribute to local melting in the shallow crust, resulting in a variety of surface expressions, such as lenticulae and microchaos (e.g., Schmidt et al. 2011; Chivers et al. 2021; Muñoz-Iglesias et al. 2019). The source of plumes is likely from brine pockets in the shallow subsurface, although sourcing from the deep ocean cannot be ruled out (Sect. 2.1). Steinbrügge et al. (2020a) show that brine could locally concentrate following impact-produced heat and melting; pressurization of such a local melt pocket could trigger the eruption of a plume. Clathrates in the crust could potentially release gas upon heating or impacting, which could also trigger the ascent of local melt by increasing buoyancy (e.g., Quick et al. 2020). Geological and thermal context provided by EIS and E-THEMIS, combined with subsurface reflections detected by REASON, is critical to determine the source depth of the material and process by which it was transferred through the crust (e.g., cryovolcanism, intrusion in faults, diapirism (Daubar et al. this collection)).

A potentially confounding parameter for interpreting the composition of Europa’s deep interior is the possible implantation of material of exogenic origin, especially sulfur released by Io. Data from PIMS may constrain our knowledge of the extent to which Io-born material (S, Na, K, and Cl) is introduced into Europa’s exosphere (Becker et al. this collection). Exogenic material may find a way to the shallow subsurface by mechanisms such as resurfacing, burying by plume ejecta, impacts, and potentially subduction (Kattenhorn and Prockter 2014). If the ice shell is permeable, the melt could be transported downward through percolation (Kalousová et al. 2014; Hesse et al. 2022). Convection could further transport exogenic material throughout the ice shell interior if resurfacing processes extend beyond the conductive lid (Howell and Pappalardo 2018). Evidence of material transport may manifest at the surface as compositional heterogeneities observed by MISE, variation in geology observed by EIS, and structural heterogeneities or water bodies in the subsurface observed by REASON.

Geological interpretation (e.g., Figueredo and Greeley 2004) and thermal–orbital evolution models (e.g., Hussmann and Spohn 2004) predict that the ice shell thickness should vary on a timescale of tens to hundreds of millions of years. Changes in ice shell thickness imply changes in ocean chemistry (Zolotov and Kargel 2009; Travis et al. 2012; Bouquet et al. 2019) and in chemical exchange between the ice shell and the ocean (Zolotov and Kargel 2009; Soderlund et al. 2020). Because the thickness of the combined water layer (liquid water plus ice) will be relatively well constrained by the moment of inertia derived from Doppler tracking (Mazarico et al. 2023) the ice shell thickness derived from combined measurements by GRS, REASON, and ECM will provide constraints on the current ocean thickness. A thin ocean would be more concentrated in the more volatile compounds that freeze below the eutectic zone. The recent generation of formation models for Europa suggest the moon’s content in volatiles beside water could be richer than considered in the past (see Becker et al. this collection). Hence, partial pressures in gas species with small kinetic diameters (e.g., CO_2_, CH_4_) could have been sufficiently high for clathrate hydrates to form in abundance (Bouquet et al. 2019). Vertical stratification resulting from changes in salt assemblages and clathrate hydrate species could potentially reveal different stages of the coupled ocean–ice system, if the contrast in dielectric properties is large enough to be resolved. Mapping of the diversity of salt and hydrate compounds from remote-sensing techniques and in-situ analysis of ejected materials and their correlation with geological units associated to different periods of the ice–ocean system cycle will provide key information on how Europa’s ocean and ice shell composition has evolved over time.

### Implications for Habitability

An environment that contains the chemical ingredients and physical conditions clement for life is defined as habitable (see Vance et al. this collection). Typically, habitable environments contain “extended regions of liquid water, conditions favorable for the assembly of complex organic molecules, and energy sources to sustain metabolism” (Des Marais et al. 2008). Assessment of habitability in the various environments of Europa requires concomitant measurements of the chemical, physical and geologic characteristics of these locales, as well as an understanding of their formation, longevity, and the interconnections between them. Through the complex array of geophysical and geochemical measurements conducted by Europa Clipper and their interpretation, interior science plays an important role in providing the scaffolding on which a carefully reasoned picture of habitability can be assembled for this enigmatic moon. Here, we address how interior science can contribute to constraining the properties and interrelationships of possible habitable environments at Europa from the bottom up, starting with the seafloor and subsurface ocean and moving through the ice–ocean interface to the ice shell and eutectic zone.

Ocean depth and salinity are important drivers for habitability, as these inform ocean composition and constrain the chemical pathways available for a putative biosphere. While Europa Clipper is not able to measure the composition of fluids directly at the seafloor, surface compositional measurements (see Becker et al. this collection) and magnetic induction measurements will be able to estimate the salinity of the ocean and provide insights to the current pH and redox state (see Vance et al. this collection). This will result in a narrower range of models for pore fluid composition by constraining the initial composition before the fluid moves downward into the mantle, as well as inform habitability investigations by constraining ocean composition and thermo-chemical evolution (Zolotov and Shock 2004; Zolotov and Kargel 2009). Crustal fluids have the capability to cycle nutrients from the deeper mantle up to the ocean and water–rock interface, providing a potential geochemical flux of biologically relevant molecules (i.e., hydrogen, methane, carbon dioxide) (Sohl et al. 2010; Vance et al. 2016; Bouquet et al. 2017). Some of these volatile species may have been transported to the surface ice over time in the form of clathrates (Bouquet et al. 2019), which Europa Clipper could identify through various lines of investigation such as composition derived from MISE, MASPEX, and SUDA observations.

If properties of the ice shell allow radar sounding to penetrate to the base of the ice shell (Sects. 1.2, 2.2), Europa Clipper may enable direct characterization of the ice–ocean interface, a potential habitable environment where strong chemical gradients may persist over geologic time (Boetius et al. 2015; Buffo et al. 2021b, 2022; Wolfenbarger et al. 2022a). Gradients in basal ice depth (e.g., basal crevasses or meridional gradients), mapped by the radar, could drive an “ice–pump” (Lewis and Perkin 1986; see also Soderlund et al. 2013; Wolfenbarger et al. 2022a) where ice that is deeper and hence at higher pressure can melt along the freezing point depression curve and re-accrete as an accumulation of individual ice crystals where the ice shell is thinner. This mechanism of ice formation is distinct from the directional thickening of the ice shell that results from cooling of the interior, and could promote heterogeneities in ice shell properties at depth that may produce radar reflections (Wolfenbarger et al. 2022a). Radar mapping of accretion and ablation are important for constraining sub-ice ocean circulation, which may be important for nutrient cycling between the ice–ocean and ocean–rock interfaces.

More broadly, constraints on, and variations in, the total ice shell thickness through radar sounding, magnetic induction, and gravity science (Sects. 1.2 and 2.2) are important for understanding potential geophysical transport processes and material exchange between the surface, ice shell, and ocean. Specifically, this may control the timescales of ice shell–ocean recycling through solid-state convection of the ice shell (e.g., Allu Peddinti and McNamara 2015) or subsumption of the ice crust (e.g., Kattenhorn and Prockter 2014). These timescales will constrain rates of delivery of key species into the ocean such as oxidants, sulfates, or nutrients from the surface (i.e., Greenberg 2010), which crucially inform Europa’s global habitability (Sect. 3.2, Vance et al. this collection).

The total ice shell thickness is, in part, a function of the planetary heat budget, which is an important parameter in constraining the energy available for life (see Vance et al. this collection). Models suggest that the rocky mantle is likely to be primarily dehydrated at the present day (Kuskov and Kronrod 2005; Castillo-Rogez and Lunine 2010), with possible cyclic upwelling of melt in the past (Travis et al. 2012; Běhounková et al. 2021; Gomez Casajus et al. 2021). However, theoretical models of rock fracturing due to thermal cooling (Vance et al. 2007) suggest that the upper tens of kilometers of the mantle may be permeable to liquid water from the ocean at the present day. If so, aqueous alteration of the near-surface mantle as well as alteration of pore fluids may occur.

Relatively large brine reservoirs (potentially ${\sim }10^{5}\text{ km}^{3}$) within the upper ∼5 km of the surface have been suggested to form chaos features on Europa’s surface (Daubar et al. this collection). They are potentially formed by injection (e.g., Michaut and Manga 2014) or the melting in situ of the ice shell (e.g., Schmidt et al. 2011). Recent models suggest that even smaller reservoirs ($\sim 10^{0}$–$10^{3}\text{ km}^{3}$) may remain liquid for ${>}10^{3}$ years (Chivers et al. 2021; Quick et al. 2021), potentially serving as transiently habitable environments (e.g., Schmidt 2020; Chivers et al. 2021) analogous to the base of terrestrial sea ice (e.g., Arrigo 2014) or ice–covered Antarctic lakes (e.g., Murray et al. 2012), where microbial communities have adapted several strategies for cold and hypersaline environments. The existence of these reservoirs may be confirmed by REASON subsurface sounding through detections of internal reflections caused by liquid water (e.g., Blankenship et al. 2009; Culha et al. 2020), or layers of hydrated salts left behind after freezing (e.g., Buffo et al. 2020; Chivers et al. 2021).

Smaller-scale reservoirs (${\sim }10^{-6}\text{ m}^{3}$), such as brine pockets within the ice shell, may also be detectable by radar sounding. Below the depth where the ice shell thermal profile exceeds the eutectic temperature (i.e., eutectic zone, see Culha et al. 2020), brine pockets are thermodynamically stable within the ice (Buffo et al. 2021a,b; Wolfenbarger et al. 2022b). However, the characteristics of the radar reflection from this dielectric contrast will be sensitive to the parameters that govern the brine volume fraction (temperature, pressure, solute composition, and salinity), as well as properties of the ice overlying the eutectic zone, which govern signal attenuation (e.g., temperature, and electrical conductivity). The eutectic zone may correspond to the boundary between a convective and conductive layer in the ice shell, the warmer basal region of a conductive ice shell, or the relatively warm head of an upwelling diapir. Radar observations of the eutectic zone will help constrain interior processes that may govern the distribution of habitable regions within Europa.

## Summary

The Level-1 science objectives for the Europa Clipper mission describe three global parameters that are of particular interest to characterize the interior of Europa: the global mean thickness of the ice shell, the mean thickness of the subsurface ocean, and the salinity of the ocean. Measuring each of these quantities to an uncertainty of ±50% or less will enable evaluation of the habitability of this ocean world.

In pursuit of these measurements, the payload on Europa Clipper includes three main investigations that are sensitive to combinations of the above parameters. The magnetic induction experiment will provide constraints on the extent and conductivity of the ocean, and the depth of the ocean below Europa’s surface. These can translate into constraints on the ice shell thickness and the salinity of the ocean. The subsurface sounding experiment characterizes the shallow subsurface and will constrain the thermophysical structure of the ice shell. These will result in an estimate of the minimum thickness of Europa’s ice shell and narrow down the thickness of the conductive portion of the ice. The tidal deformation measurements are sensitive to a combination of the thickness of the ice shell and its rigidity, providing an additional independent constraint on the thickness of the elastic portion of the ice shell.

While each of these investigations alone will reveal critical information about the interior of Europa, none of them can provide a comprehensive view. Together, they support each other and are highly complementary. Combining multiple datasets is a powerful way of characterizing the interior of Europa and habitability of its subsurface ocean. These investigations are supplemented by a variety of ancillary investigations that can further reduce ambiguity and provide a unique view of the interior of one of the most compelling ocean worlds known to planetary science.
